# Modification of a Constitutive to Glucose-Responsive Liver-Specific Promoter Resulted in Increased Efficacy of Adeno-Associated Virus Serotype 8-Insulin Gene Therapy of Diabetic Mice

**DOI:** 10.3390/cells9112474

**Published:** 2020-11-13

**Authors:** Kian Chuan Sia, Zhen Ying Fu, Roy Y. Calne, Amit C. Nathwani, Kok Onn Lee, Shu Uin Gan

**Affiliations:** 1 Department of Surgery, National University of Singapore, Singapore 117597, Singapore; sursiak@nus.edu.sg (K.C.S.); surfz@nus.edu.sg (Z.Y.F.); ryc1000@medschl.cam.ac.uk (R.Y.C.); 2 Department of Surgery, University of Cambridge, Cambridge CB2 0QQ, UK; 3 Department of Haematology, UCL Cancer Institute, London WC1E 6DD, UK; amit.nathwani@ucl.ac.uk; 4 Department of Medicine, National University of Singapore, Singapore 119228, Singapore; mdcleeko@nus.edu.sg

**Keywords:** liver-specific glucose-responsive promoter, adeno-associated virus serotype 8 (AAV8), diabetes gene therapy, long-term basal insulin expression, glucose responsive element (GlRE), albumin enhancer (3′iALB)

## Abstract

We have previously used a hepatotropic adeno-associated viral (AAV) vector with a modified human insulin gene to treat diabetic mice. The HLP (hybrid liver-specific promoter) used was constitutively active and non-responsive to glucose. In this study, we examined the effects of addition of glucose responsive elements (R3G) and incorporation of a 3′ albumin enhancer (3′iALB) on insulin expression. In comparison with the original promoter, glucose responsiveness was only observed in the modified promoters in vitro with a 36 h lag time before the peak expression. A 50% decrease in the number of viral particles at 5 × 10^9^ vector genome (vg)/mouse was required by AAV8-R3GHLP-hINSco to reduce the blood sugar level to near normoglycemia when compared to the original AAV8-HLP-hINSco that needed 1 × 10^10^ vg/mouse. The further inclusion of an 860 base-pairs 3′iALB enhancer component in the 3′ untranslated region increased the in vitro gene expression significantly but this increase was not observed when the packaged virus was systemically injected in vivo. The addition of R3G to the HLP promoter in the AAV8-human insulin vector increased the insulin expression and secretion, thereby lowering the required dosage for basal insulin treatment. This in turn reduces the risk of liver toxicity and cost of vector production.

## 1. Introduction

Insulin is essential in the treatment of type 1 diabetes, and a key component in the treatment of the more common type 2 diabetes patient. Two types of insulin are used in both type 1 and type 2 diabetes; a long acting or “basal” insulin, usually injected once a day, and rapid and short acting insulin, usually injected multiple times a day prior to mealtimes. The duration of action of the basal insulins ranges from 16 h in detemir [1] to near 48 h in degludec [2]. Having a basal level insulin is thought to be important in preventing the development of several long-term complications in diabetes [3,4].

Our previous studies reported the use of an adeno-associated virus serotype 8 (AAV8) viral vector that expresses furin cleavable codon optimized human proinsulin [5,6] driven by a constitutive liver specific HLP (hybrid liver-specific promoter) promoter [7]. When the vector was systemically injected and specifically expressed in the liver of streptozotocin (STZ)-induced diabetic mice [5] and non-obese diabetic (NOD) mice [6], we observed a decrease in the hyperglycemia and restoration of weight in the treated mice. We have also reported preliminary results of similar study in a naturally occurring diabetic dog [8].

In this paper, we aimed to further reduce the requirement of virus for in vivo administration of AAV8 vector by the following two strategies: (1) Enhance the HLP promoter to make it glucose responsive by incorporating 3 copies of glucose responsive element (GlRE) from liver pyruvate kinase (LPK) promoter [9] at 5′ end of HLP promoter. GlRE consisted of carbohydrate response element (ChoRE) and DR1 sites that are crucial for successful conversion of HLP promoter to glucose responsive promoter. The ChoRE consisted of two E-Boxes sites for transcription factors, i.e., the carbohydrate response element binding protein (ChREBP) to bind. The distance between these E-box must be exactly 5 nucleotides for proper function of glucose-induced gene expression [10]. The binding of ChREBP on ChoRE in LPK promoter has been demonstrated to play a main role in glucose-induced gene expression [11]. DR1 site further enhanced the glucose responsive gene expression via the binding and interaction of homodimer HNF4α nuclear transcription factor [12] and the co-activator CBP [13] with ChREBP/Mlx heterodimer [14] when the glucose level is high. On the other hand, in a low glucose environment, the binding of COUP-TFII [15,16] and FXR [17] transcriptional repressor proteins onto the DR1 site inhibits the downstream gene expression. More detailed description on cross-regulation of hepatic glucose metabolism via ChREBP and nuclear receptor was reviewed elsewhere [18]. (2) Increase the expression level of human insulin by inserting 3′ untranslated region (3′UTR) of albumin gene with intron 14, in short 3′iALB [19,20] as enhancer at 3′ end of proinsulin gene before poly A sequences. 3′iALB could potentially increase the production of desired protein through improving the stability, localization and translation of mRNA [21].

## 2. Materials and Methods

### 2.1. Plasmid DNA Constructs

**Figure 1:**
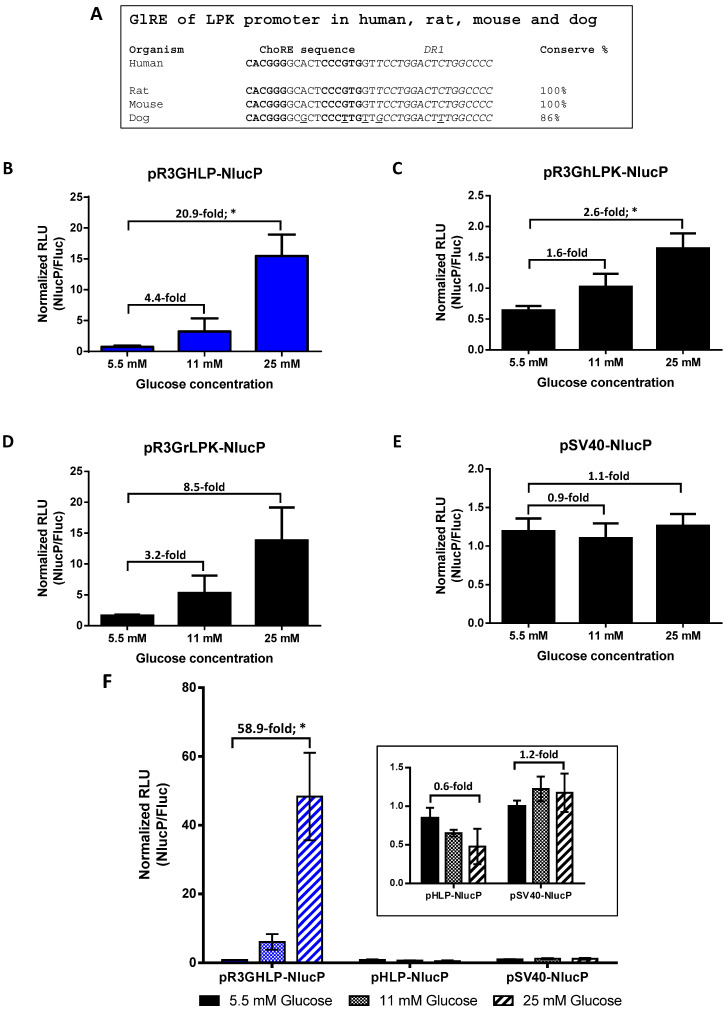
Effects of conserved glucose responsive elements in liver pyruvate kinase (LPK) promoter, glucose responsive elements (R3G) in converting constitutive human liver-specific promoter to glucose responsive human liver-specific promoter. (**A**) Sequence alignment of glucose responsive element (GlRE) sequences from LPK promoters between human, rat, mouse and dog. The conserved regions consist of carbohydrate response element (ChoRE) sequence and DR1 site. (**B**) Glucose responsive gene expression by R3GHLP glucose responsive human liver-specific promoter. (**C**) R3GhLPK and (**D**) R3GrLPK with human and rat LPK minimal promoters, respectively, were used as positive controls. (**E**) SV40 promoter was used as negative control. (**F**) Comparison of glucose responsiveness and gene expression level of R3GHLP glucose responsive human liver-specific promoter versus HLP constitutive human liver-specific promoter. Freshly isolated primary rat hepatocytes (rHeps) culture were co-transfected with experimental plasmid expressing PEST-destabilized nanoLuc luciferase (NlucP) reporter plasmid and pSV40-Luc2 expressing firefly luciferase (Fluc) constitutively expressed control reporter plasmid. For each sample, NlucP activity was normalized to Fluc activity from pSV40-Luc2 regulated by SV40 constitutive promoter. Glucose were added to a final concentration of 5.5 mM, 11 mM, or 25 mM. Data were presented as mean ± standard error of mean (s.e.m.) *n* = 3. Statistical significances were determined by Student’s unpaired *t*-test where * indicates *p* < 0.05 and considered statistically significant.

Schematic diagrams of all the cloning strategies were illustrated in Supplementary Figure S1A–N. Phylogenetically conserved GlRE DNA sequence (Figure 1A) was synthesized (Integrated DNA Technology, Coralville, IA, USA) in reverse orientation with SpeI site at 5′ end and NheI followed by XbaI sites at 3′ end.

The synthesized DNA was cloned in pIDT-SMART-GlRE shutter vector. To multimerize the reverse GlRE sequence, single reverse GlRE sequence was excised from the shutter vector using SpeI/XbaI and inserted into the compatible end of same linearized shutter vector digested with NheI. This cloning method was repeated again to generate pIDT-SMART-3xGlRE (Supplementary Figure S1A) containing 3 copies of reverse GlRE (in short R3G). R3G fragment was then released using HindIII/SpeI and subcloned into the HindIII/NheI (compatible end with SpeI) sites of pNL1.2[*NlucP*] (Promega, Madison, WI, USA). This was followed by the insertion of HLP promoter downstream of the R3G using the NheI/HindIII sites to generate pR3GHLP-NlucP (Supplementary Figure S1B). Similar strategies were used to generate pR3GhLPK-NlucP (Supplementary Figure S1C) and pR3GrLPK-NlucP (Supplementary Figure S1D). Negative control pSV40-NlucP (Supplementary Figure S1E) was generated by inserting the SV40 promoter from pGL3-Control (Promega) into pNL1.2[*NlucP*] (Promega) using NheI and HindIII sites. Normalization control pSV40-Luc2 (Supplementary Figure S1F) was generated by swapping the NlucP in pSV40-NlucP with Luc2 gene from pGL4.10[*luc2*] (Promega) using HindIII and XbaI sites. For pHLP-NlucP (Supplementary Figure S1G), HLP promoter from previously generated pAAV-HLP-Luc [22] was released with SpeI/HindIII and subcloned into pNL1.2[*NlucP*] using NheI (compatible end with SpeI) and HindIII sites. pAAV-HLP-hINSco, an AAV plasmid construct containing HLP promoter driving the expression of codon optimized furin cleavable human proinsulin gene (denoted as hINSco), was previously generated [5]. For pAAV-R3GHLP-hINSco (Supplementary Figure S1H) construction, HLP promoter in pAAV-HLP-hINSco was swapped with R3GHLP promoter from pAAV-R3GHLP-Luc (see below for its construction) using HincII and HindIII to generate pAAV-R3GHLP-hINSco. The enhancer 3′iALB was amplified from normal human genomic DNA according to similar strategy as described by Wooddell et al. [20]. The PCR fragment of 3′iALB was cloned into a shutter vector generating 3′iALB shutter vector (Supplementary Figure S1I) where 3′iALB fragment could be removed using EcoRI and XbaI. The excised 3′iALB fragment was then klenow-blunted and subcloned into klenow-blunted FseI-linearized pR3GHLP-NlucP to generated both pR3GHLP-NlucP-e(+) (Supplementary Figure S1J) and pR3GHLP-NlucP-e(−) (Supplementary Figure S1K). To construct pAAV-R3GHLP-Luc (Supplementary Figure S1L), pAAV-HLP-Luc was first digested with SpeI followed by klenow blunt and HindIII digestion to generate promoterless empty AAV plasmid with blunt and HindIII sticky end. R3GHLP promoter was removed from pR3GHLP-NlucP using KpnI followed by klenow blunt and HindIII digestion. The R3GHLP promoter was then sticky-blunt end ligated into promoterless AAV plasmid to generate pAAV-R3GHLP-Luc. Similar to above mentioned strategy, blunt ended 3′iALB fragment was inserted into the Klenow-treated BglII-linearized pAAV-R3GHLP-Luc to generated pAAV-R3GHLP-Luc-e(+) (Supplementary Figure S1M). To generate pAAV-R3GHLP-hINSco-e(+) (Supplementary Figure S1N), luciferase reporter gene in pAAV-R3GHLP-Luc-e(+) was removed using ApaI (followed by klenow blunt) and SpeI and replaced with hINSco gene from pAAV-HLP-hINSco (excised using XhoI followed by klenow blunt and SpeI digestion). 

### 2.2. Primary Rat Hepatocyte Cultures

Freshly isolated primary rat hepatocytes (rHeps) obtained from male Wistar rats (HanTac:WH) were kindly provided by Professor Hanry Yu from Department of Physiology, National University of Singapore. Upon receiving the rHeps, the cells were immediately cultured with complete Williams’ E Medium (WEM) (Sigma-Aldrich, St. Louis, MO, USA) [supplemented with 1 mg/mL bovine serum albumin (Sigma-Aldrich), 100 U/mL penicillin (PAA Laboratories Inc., Etobicoke, ON, Canada), 100 µg/mL streptomycin (PAA laboratories Inc.), 2 mM L-glutamine (Gibco, Grand Island, NY, USA), 100 nM dexamethasone (Sigma-Aldrich), 0.5 µg/mL recombinant human insulin (Sigma-Aldrich), and 50 ng/mL linoleic acid (Sigma-Aldrich)]. Cells were seeded at 1.2 × 10^5^ cells/cm^2^ in 100 µL of complete WEM in rat-tail type I collagen (Gibco) coated 96-well plates.

### 2.3. Transfection of Primary Rat Hepatocytes

Primary rat hepatocytes were transfected with total of 100 ng of desired plasmids (i.e., 25 ng of NlucP plasmid driven by promoter of interest, and 75 ng of pSV40-Luc2 plasmid (Fluc)) 4 h post seeding in 96-well plates using 0.6 µL lipofectamine 3000 and 0.2 µL of P3000 according to manufacturing protocol (Invitrogen, Carlsbad, CA, USA). Cells were transfected in complete WEM (USBiological, Salem, MA, USA) containing 5.5 mM glucose (Sinopharm Chem, Shanghai, China) instead of the usual 11 mM glucose, for subsequent glucose induction studies.

### 2.4. Luciferase Reporter Assay for Studying Glucose Responsive Promoter

After overnight transfection, cells were replenished with 80 µL of complete WEM containing either 5.5, 11, or 25 mM glucose. These cells were continuously cultured for another 24 h before subjected to Nano-Glo Dual-Luciferase reporter assay according to manufacturer’s protocol (Promega). The emitted light unit in count per second (CPS) was measured using Victor 3 multilabel plate reader (PelkinElmer, Waltham, MA, USA).

### 2.5. Packaging, Purification and Titration of AAV8 Viral Vector

All AAV8 vector particles (single-stranded AAV8) were made by the 293T transient triple transfection method with linear polyethylenimine, MW 25,000 (Polysciences, Warrington, PA, USA) as previously described using an adenoviral helper plasmid (HGT1), a chimeric AAV2 Rep-8Cap packaging plasmid (pAAV2-8) and AAV plasmid [5] with the respective promoters and genes flanked inverted terminal repeats from AAV2. Serotype 8 capsid pseudotyped viral particles were purified by the previously described iodixanol density gradient method [23]. The extracted vector particles were further purified from iodixanol contamination and concentrated using Amicon Ultra 4 100K filter device (Millipore, Billerica, MA, USA) with PBS before aliquot in 50 µL per tube and stored in −80 °C. Vector particles were titrated by quantitative PCR as described previously [24].

### 2.6. Animal Work

All animal experiments were performed according to the guidelines and protocols approved by the Institutional Animal Care and Use Committee (IACUC) of the National University of Singapore (protocol no. 2013-05448). The NOD.cg-PrkdcscidIl2rgtm1Wjl/SzJ (NSG) breeders (The Jackson Laboratory, Bar Harbor, ME, USA) were bred and maintained in the specific pathogen-free facility within the university. The mice were subjected to regular 12 h dark/light cycles and provided with ad libitum of normal feed and water unless otherwise stated. Throughout this study, 8–12 weeks old male NSG mice with entry weight ranging from 26–30 g were used for diabetes induction. Multiple low-dose injection of STZ was used to induce diabetes, resulting in the ablation of pancreatic beta-cells and insulin deficiency. Diabetes was induced with 5 consecutive daily intraperitoneal injections of 40 mg/kg STZ (Sigma-Aldrich) according to The Jackson Laboratory’s data. Body weight was measured and blood was obtained via the tail vein for blood glucose measurements with a glucometer (Roche Diagnostics GmbH, Mannheim, Germany). Mice that maintained their blood glucose levels at >20 mM for 4 consecutive days were considered diabetic. We performed our experiments by randomly assigning STZ induced diabetic mice to receive different doses and types of AAV8. In order to reduce variation between different batches of virus preparations, AAV8s used in same experiment were titered at the same time before the experiment. For AAV8-R3GHLP-hINSco-e(+) and its control AAV8-HLP-hINSco, the AAV8s were packaged, purified and titered at the same time before the experiment. The desired AAV8 vectors were subsequently injected into the diabetic mice via the tail vein with specified numbers of vector genome per mouse (vg/mouse). Blood was obtained on the indicated time points post AAV8 injection and at the end point prior to euthanasia. Mice that became moribund or severely underweight were euthanized by hypoxia using carbon dioxide or isoflurane overdose followed by cervical dislocation. All efforts were made to minimize suffering.

### 2.7. AAV Viral Genome Copies Quantification

Total genomic DNA from the livers of treated mice were extracted using AllPrep DNA/RNA mini kit according to manufacturing protocol (QIAGEN, Valencia, CA, USA). AAV viral genome copy number was determined by real-time qPCR using Rotor-Gene 3000 (Corbett Research, Sydney, Australia) and Rotor-Gene SYBR Green PCR Kit (QIAGEN). The primers were designed against the HLP promoter and normalized with mouse GAPDH housekeeping gene to calculate AAV genome copies/mouse cell.

### 2.8. C-Peptide ELISA

Serum human C-peptide at endpoint was measured using an ELISA kit for human C-peptide (Millipore) according to manufacturing instruction. This kit does not cross react with mouse C-peptide.

### 2.9. Fasting and Intraperitoneal Glucose Tolerant Test (IPGTT)

Fasting of the treated and control animals were carried out by withdrawing the food and bedding from cages, leaving the water bottle behind. Blood glucose readings were obtained at the indicated time points by glucometer. For IPGTT, mice were fasted for 4 to 6 h before subjected to intraperitoneal injection with 2 g/kg of 20% glucose solution in PBS. Blood glucose levels were continuously monitored at the indicated time points.

### 2.10. Statistical Analyses

The number of animals used in each group was indicated in the respective figure legends. All values are expressed as mean ± s.e.m. Statistical significance for studies involving two groups were determined by Student’s unpaired *t*-test whereas statistical significance for studies involving more than two factors were determined by two-way ANOVA where ns indicated not significant, * indicates *p* < 0.05 and considered statistically significant, ** indicates *p* < 0.01 and considered very significant, *** indicates *p* < 0.001 and considered extremely significant and **** indicates *p* < 0.0001 and considered extremely significant.

## 3. Results

### 3.1. Generation of Novel Glucose Responsive Promoter from Constitutive Hybrid Liver-Specific Promoter for Diabetic Gene Therapy

To generate the glucose responsive promoter, we multimerized three copies of phylogenetically conserved glucose responsive element (GlRE) from liver pyruvate kinase (LPK) promoter (Figure 1A) and cloned it, in reverse orientation, upstream of hybrid liver-specific HLP promoter.

The glucose responsive hybrid liver-specific promoter was denoted as R3GHLP. R3GhLPK and R3GrLPK consisting of human LPK and rat LPK promoter, respectively, were also generated to serve as positive controls since the GlRE elements were originally from the LPK promoters.

The glucose responsiveness of these hybrid promoters driving the expression of nanoLuc-PEST (NlucP) gene was evaluated in freshly isolated rHeps cultured in vitro. The NlucP reporter gene was used to ensure promoters’ transcriptional activities could be closely and accurately reported. The inclusion of PEST peptide sequence targeted the nanoLuc for degradation and reduce its half-life from more than 6 h to 10 to 30 min. This prevented the intracellular accumulation of nanoLuc which increased signal-to background ratios [25].

In Figure 1B, R3G successfully transformed the constitutive HLP liver-specific promoter to glucose responsive liver-specific promoter with 4.4-fold increase at 11 mM glucose and 20.9-fold increase at 25 mM glucose when compared to gene expression level at 5.5 mM glucose. Additionally, R3GHLP promoter also showed significant increase in gene induction fold especially at high glucose concentration when compared to both R3GhLPK (Figure 1C) and R3GrLPK (Figure 1D) positive controls. Such glucose responsive effect was not observed in SV40 constitutive promoter that served as negative control (Figure 1E). When comparing the expression levels between R3GHLP and HLP promoters at 5.5 mM, 11 mM and 25 mM glucose in rHeps culture (Figure 1F), R3GHLP promoter consistently showed enhanced gene expression level in a glucose dependent manner, whereas the HLP promoter has similar basal luciferase expression at 5.5 mM but was not glucose responsive.

### 3.2. Treatment Efficacy of Diabetic Mouse with Constitutive and Glucose Responsive Promoter Driving the Expression of Human Insulin

**Figure 2:**
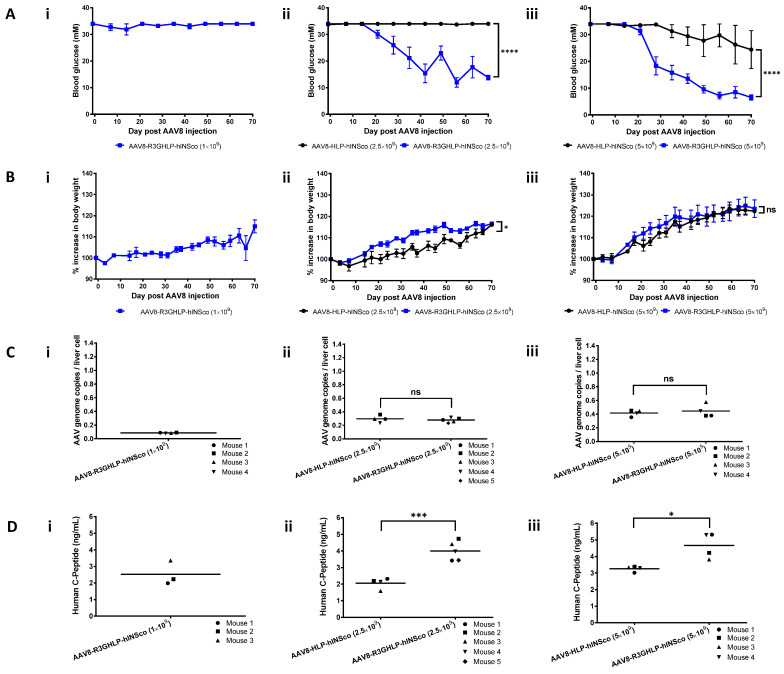
Treatment efficacy of AAV8-R3GHLP-hINSco on streptozotocin (STZ)-induced diabetic NOD.cg-PrkdcscidIl2rgtm1Wjl/SzJ (NSG) mice. Treatment efficacy of control non-glucose responsive adeno-associated virus serotype 8 (AAV8) vector, AAV8-HLP-hINSco and glucose responsive AAV8 vector, AAV8-R3GHLP-hINSco at (**i**) 1 × 10^9^ (AAV8-R3GHLP-hINSco only), (**ii**) 2.5 × 10^9^ and (**iii**) 5 × 10^9^ vector genome per mouse (vg/mouse). (**Ai**–**iii**) Blood glucose level post AAV8 injection. (**Bi**–**iii**) Percentage increase in body weight post AAV8 injection. (**Ci**–**iii**) Corresponding AAV genome copies per liver cell at endpoint. (**Di**–**iii**) Corresponding serum human C-peptide concentration at endpoint. Data were presented as mean±s.e.m. *n* = 4–5. Statistical significances for (**A**,**B**) were determined by two-way ANOVA where ns indicated not significant, * indicates *p* < 0.05 and considered statistically significant and **** indicates *p* < 0.0001 and considered extremely significant. Statistical significances for (**C**,**D**) were determined by Student’s unpaired *t*-test where ns indicated not significant, * indicates *p* < 0.05 and considered statistically significant and *** indicates *p* < 0.0001 and considered extremely significant.

To evaluate the ability of R3GHLP promoter to treat diabetic mice, the promoter was cloned into AAV plasmid vector to drive the expression of codon optimized furin-cleavable human proinsulin (hINSco) gene and packaged into AAV8 viral particles. The efficacy of diabetes treatment was monitored for 70 days and shown in Figure 2.

AAV8-R3GHLP-hINSco injected with 1 × 10^9^ vg/mouse was not effective in lowering the blood glucose nor significantly improved the body weight (Figure 2Ai,Bi). Mice injected with AAV8-R3GHLP-hINSco was able to lower the blood glucose significantly compared to those injected with AAV8-HLP-hINSco at doses of 2.5 × 10^9^ (Figure 2Aii,Bii) and 5 × 10^9^ (Figure 2Aiii,Biii) vg/mouse.

AAV genome copy numbers per liver cell for each group of mice (Figure 2Ci–iii) confirmed that similar amount of AAV8 were delivered into liver cells via systemic administration, thereby excluding the possibility that the differential efficacy was due to inconsistent gene delivery. ELISA quantification of human C-peptide in serum obtained at endpoint further confirmed that AAV8-R3GHLP-hINSco was more effective than AAV8-HLP-hINSco in reducing the blood glucose level as well as increasing the body weight via higher human insulin expression level in liver (Figure 2Di–iii).

**Figure 3:**
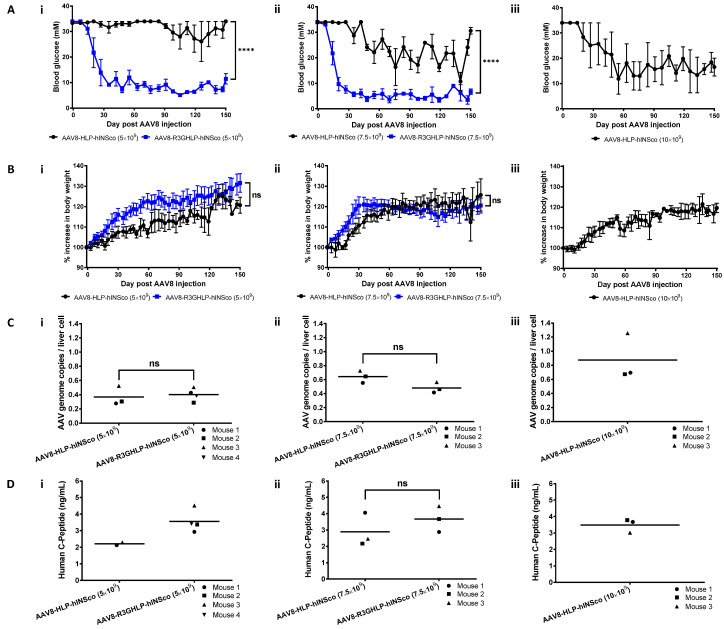
Treatment efficacy and tolerability of AAV8-R3GHLP-hINSco on STZ-induced diabetic NSG mice at higher dose. Control non-glucose responsive AAV8 vector, AAV8-HLP-hINSco and glucose responsive AAV8 vector, AAV8-R3GHLP-hINSco were injected at various doses at (**i**) 5 × 10^9^, (**ii**) 7.5 × 10^9^ and (**iii**) 10 × 10^9^ (AAV8-HLP-hINSco only) vg/mouse. (**Ai**–**iii**) Blood glucose level post AAV8 injection. (**Bi**–**iii**) Percentage increase in body weight post AAV8 injection. (**Ci**–**iii**) Corresponding AAV genome copies per liver cell at endpoint. (**Di**–**iii**) Corresponding serum human C-peptide concentration at endpoint. Data were presented as mean ± s.e.m. *n* = 3–4. Statistical significances for (**A**,**B**) were determined by two-way ANOVA where ns indicated not significant and **** indicates *p* < 0.0001 and considered extremely significant. Statistical significances for (**C**,**D**) were determined by Student’s unpaired *t*-test where ns indicated not significant.

In a subsequent experiment we repeated the effective dose of 5 × 10^9^ vg/mouse (Figure 3Ai,Bi), with additional groups of higher doses at 7.5 × 10^9^ (Figure 3Aii,Bii) and 10 × 10^9^ (Figure 3Aiii,Biii) vg/mouse to determine the maximum insulin vector dose that the mice can tolerate.

We also monitored the efficacy over a longer period of 150 days. The vector genome per mouse of AAV8-R3GHLP-hINSco was reproducibly found to be more efficient than AAV8-HLP-hINSco in normalizing the blood glucose of diabetic mice. The blood glucose decreased to the normal range (Figure 3Ai) and body weight showed increment (Figure 3Bi) in mice injected with 5 × 10^9^ vg/mouse of AAV8-R3GHLP-hINSco. On the other hand, mice injected with 5 × 10^9^ vg/mouse AAV8-HLP-hINSco dose remained diabetic but showed slow increase in body weight similar to the AAV8-R3GHLP-hINSco group (Figure 3Ai,Bi). The AAV8-HLP-hINSco needed at least 10 × 10^9^ vg/mouse to achieve glucose lowering similar effects (Figure 3Aiii,Biii). The mice injected with 7.5 × 10^9^ vg/mouse of AAV8-R3GHLP-hINSco experienced hypoglycemia episodes (Figure 3Aii). AAV8-R3GHLP-hINSco at 10 × 10^9^ vg/mouse dose was not tested in this study as our preliminary data suggested that this dose was not safe and the mice died due to hypoglycemia (data not shown). Corresponding AAV genome copies per liver cell and ELISA quantification of human C-peptide in serum obtained at endpoint for each group of mice were reported in Figure 3Ci–iii and Figure 3Di–iii, respectively.

A higher dose (at least 10 × 10^9^ vg/mouse) was required for AAV8-HLP-hINSco (Figure 3Aiii) to achieve a similar level of glycemic control by AAV8-R3GHLP-hINSco at 5 × 10^9^ vg/mouse (Figure 3Ai). The blood sugar of mice injected with 7.5 × 10^9^ vg/mouse of AAV8-HLP-hINSco (Figure 3Aii) was partially corrected and fluctuated between normal and diabetic range (10–30 mM).

### 3.3. Comparison Between Different Promoters for Fasting Effect and IPGTT

**Figure 4:**
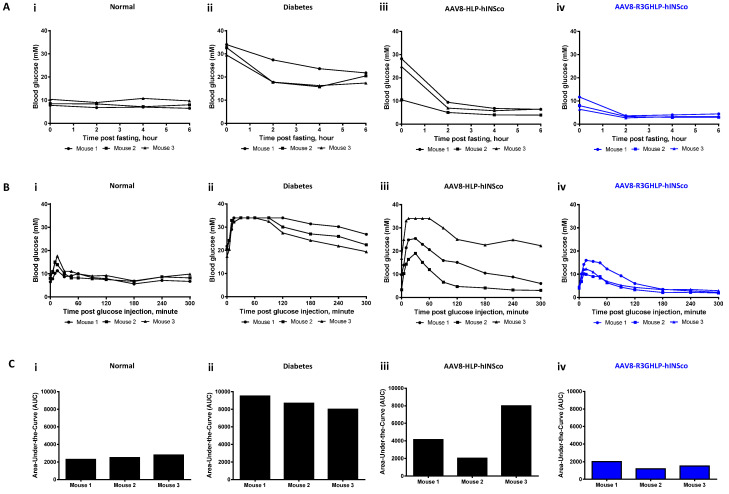
Fasting blood glucose and intraperitoneal glucose tolerance test (IPGTT) of STZ-induced diabetic NSG mice treated with optimum dose of AAV8-R3GHLP-hINSco. Comparison of (**A**) fasting blood glucose (performed at day 73 post AAV8 injection) and (**B**) IPGTT (performed at day 92 post AAV8 injection) for (**i**) normal mice, (**ii**) diabetic mice, (**iii**) AAV8-HLP-hINSco (7.5 × 10^9^ vg/mouse) treated mice, and (**iv**) AAV8-R3GHLP-hINSco (5 × 10^9^ vg/mouse) treated mice. Normal and diabetic mice were included as controls. (**Ci**–**iv**) Corresponding area-under-the-curve (AUC) of IPGTT. Data points of individual mouse were shown. *n* = 3.

Effect of fasting (Figure 4A) and intraperitoneal glucose tolerance test (IPGTT; Figure 4B) were studied on normal (Figure 4Ai,Bi) and diabetic (Figure 4Aii,Bii) mice to serve as controls for comparison with diabetic mice injected with AAV8-HLP-hINSco (7.5 × 10^9^ vg/mouse; Figure 4Aiii,Biii) and AAV8-R3GHLP-hINSco (5 × 10^9^ vg/mouse; Figure 4Aiv,Biv).

These treated groups were chosen for comparison as they showed similar blood glucose range between 3 to 7 mM after fasting (Figure 4Aiii–iv). The blood glucose levels of healthy mice were stable in normal range between 6.5 to 9.7 mM glucose after 6 h of fasting (Figure 4Ai) whereas in diabetic mice the blood glucose remained at hyperglycemic range of above 20 mM glucose (Figure 4Aii). For AAV8-HLP-hINSco (7.5 × 10^9^ vg/mouse), two mice were diabetic at the start of the experiment (24.8 and 28.2 mM blood glucose), but the blood glucose level rapidly dropped and stabilized at levels between 3.9 to 6.8 mM similar to normal (6.5 to 10.8 mM) and AAV8-R3GHLP-hINSco (2.9–4.4 mM) groups (Figure 4Aiii). The fasting blood glucose levels of mouse 2 of AAV8-HLP-hINSco and AAV8-R3GHLP-hINSco treated groups were in the hypoglycemia range of less than 5 mM blood glucose (Figure 4Aiii–iv) while healthy mice were capable of maintaining their blood glucose within normal range (Figure 4Ai). Except for the starting blood glucose level, no apparent difference was observed for the fasting blood glucose between the mice injected with AAV8-HLP-hINSco (Figure 4Aiii) and AAV8-R3GHLP-hINSco (Figure 4Aiv).

IPGTT in normal mice showed that intraperitoneal injected blood glucose was rapidly normalized (within 30 min) and maintained in normal range for up to 300 min (Figure 4Bi). In diabetic mice, impaired IPGTT result was observed (Figure 4Bii). Mice treated with AAV8-HLP-hINSco and AAV8-R3GHLP-hINSco showed similar pattern of glucose responsiveness albeit returning to the blood glucose level prior to IPGTT at different time points after glucose injections (Figure 4Biii–iv). The results obtained from mice injected with AAV8-HLP-hINSco (7.5 × 10^9^ vg/mouse) were more variable. Mouse 1 had fasting blood glucose of 9.7 mM prior to IPGTT, and blood glucose was normalized after 240 min. The fasting blood glucose of mouse 2 was 3.3 mM and it took 90 min for the level to normalize (Figure 4Biii). The fasting blood glucose of mouse 3 was 16.8 mM prior to the IPGTT, which was higher than 5.8 mM observed in fasting experiment performed on another day (Figure 4Aiii). This diabetic range of fasting blood glucose before IPGTT led mouse 3 to exhibit glucose intolerance similar to diabetic mice. Fluctuation of fasting blood glucose in mouse 3 may be caused by approximately 20% higher body weight (32.1 g) before AAV8 injection when compared to mouse 1 (26.2 g) and 2 (25.6 g) (Supplementary Figure S2A). It also had higher blood glucose measurement as shown in Supplementary Figure S2B. However, result of AAV genome copies per liver cell (Figure 3Cii; AAV8-HLP-hINSco 7.5 × 10^9^ vg/mouse) and human C-peptide level (Figure 3Dii; AAV8-HLP-hINSco 7.5 × 10^9^ vg/mouse) revealed that mouse 3 contained similar number of AAV genome copies per liver cell and human C-peptide level comparable to mouse 2 suggesting that glucose intolerance in mouse 3 was not due to insufficient expression of human insulin due to fix dose of AAV injection. Although large variation was observed between 3 individual mice, the result suggested that HLP liver-specific promoter expressing insulin gene was able to normalize the blood glucose during IPGTT when the fasting blood glucose was within the normal range at the start of the experiment. With AAV8-R3GHLP-hINSco (5 × 10^9^ vg/mouse) containing glucose responsive promoter, two mice (4 mM fasting blood glucose before IPGTT for both mice) showed IPGTT blood glucose normalization in less than 60 min and one mouse (4.6 mM fasting blood glucose before IPGTT) within 120 min (Figure 4Biv). The corresponding area-under-the-curve (AUC) for all tested groups in IPGTT were also calculated (Figure 4Ci–iv) to indicate the glycemic index of each individual mouse. The results presented here are qualitative in nature, and we would need to repeat the experiment with more animals per group for statistical analyses.

### 3.4. Enhanced R3GHLP Promoter Activity in Glucose Dependent Manner with 3′iALB Enhancer

**Figure 5:**
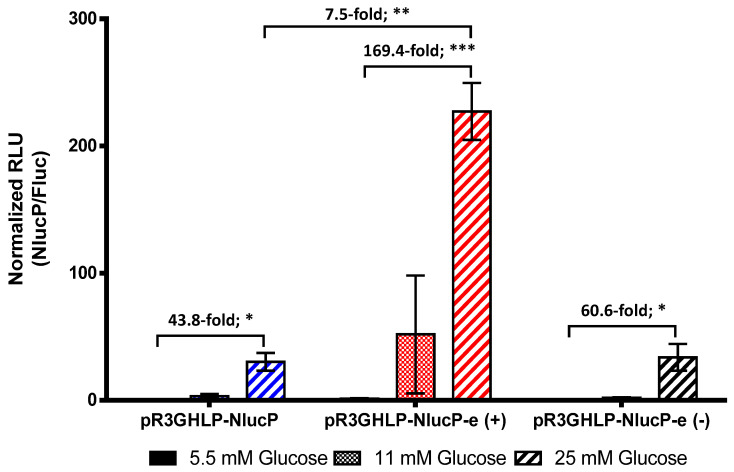
Incorporation of human albumin 3′untranslated region with intron, 3′ albumin enhancer (3′iALB) for improved glucose responsiveness and enhanced gene expression level. Glucose responsive gene expression conferred by pR3GHLP-NlucP-e (3′iALB denoted as “e” in plasmid’s name) at various glucose concentration at 5.5, 11, and 25 mM. The expression level of R3GHLP promoter with 3′iALB at forward direction, pR3GHLP-NlucP-e(+) and reverse direction, pR3GHLP-NlucP-e(−) were compared. The experiment was performed in vitro with freshly isolated primary rHeps culture where plasmids were transfected using lipofectamin 3000. NlucP was used as reporter gene and the expression levels were normalized to Fluc activities from co-transfected pSV40-Luc2 constitutively expressed control reporter plasmid. All data were presented as mean ± s.e.m. *n* = 3. Statistical significances were determined by Student’s unpaired *t*-test where * indicates *p* < 0.05 and considered statistically significant, ** indicates *p* < 0.01 and considered very significant and *** indicates *p* < 0.001 and considered extremely significant.

In order to further improve the efficacy of R3GHLP vector, 3′ UTR of human albumin gene with intron 14, denoted as 3′iALB [19,20] was inserted at 3′ end of reporter gene. Plasmid ended with “-e” indicated plasmid that containing 3′iALB. When transfected to rHeps, glucose responsiveness of R3GHLP promoter (5.5 mM versus 25 mM glucose) was further enhanced from 44-fold to 169-fold (Figure 5) with inclusion of 3′iALB.

In addition, the total gene expression level was also increased 7.5-fold with 3′iALB in complete medium containing 25 mM glucose. Interestingly, when the 3′iALB was inserted at reverse orientation, i.e., pR3GHLP-NlucP-e(−), the enhancer function was not observed and the glucose responsiveness was only slightly increased from 44-fold to 61-fold with no significant increase in overall gene expression level. This result underscored the importance of direction when using 3′iALB as enhancer.

The 3′iALB enhancer was subsequently subcloned into an existing AAV plasmid vector backbone consisting the HLP promoter driving a firefly luciferase (Fluc) reporter gene [22]. The transfection of the plasmid into rHeps indicated that 3′iALB enhancer remained functional and showed increase in both glucose responsiveness fold and overall luciferase activity in AAV construct (Supplementary Figure S3A). The kinetics of the gene expression upon adding the glucose (from 5 mM to 25 mM glucose) and withdrawal of glucose (from 25 mM to 5 mM glucose) was also evaluated (Supplementary Figure S3B). The result demonstrated that full glucose responsiveness of R3GHLP promoter with 3′iALB enhancer was maintained in AAV plasmid vector. Upon increasing the glucose from 5.5 mM to 25 mM, the gene expression level continuously increased and reached its maximum at about 36 h post induction. In contrast, when the glucose was reduced from 25 mM to 5.5 mM, gene expression also started to reduce continuously until the end of experiment at 48 h.

**Figure 6:**
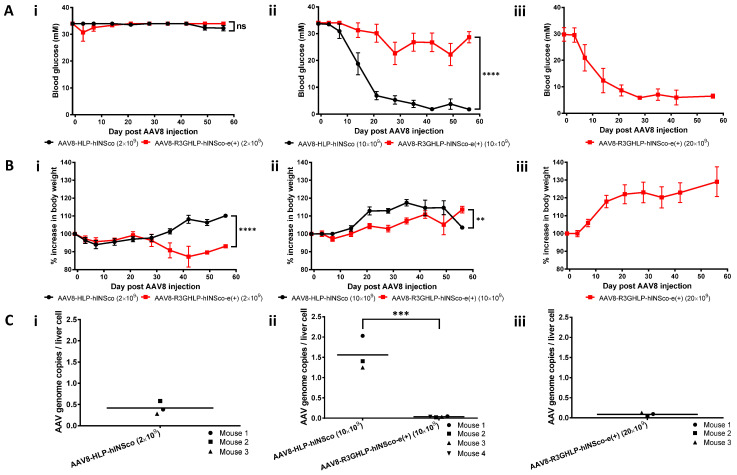
Treatment efficacy of AAV8-R3GHLP-hINSco-e(+) on STZ-induced diabetic NSG mice. Treatment efficacy of control non-glucose responsive AAV8 vector, AAV8-HLP-hINSco and glucose responsive AAV8 vector with 3′iALB enhancer, AAV8-R3GHLP-hINSco-e(+) at various doses at (**i**) 2 × 10^9^, (**ii**) 10 × 10^9^ and (**iii**) 20 × 10^9^ (AAV8-R3GHLP-hINSco-e(+) only) vg/mouse. AAV8-HLP-hINSco at 20 × 10^9^ vg/mouse was not included as this high AAV8 dose was previously found to cause severe hypoglycemia with high incidence of mortality. (**Ai–iii**) Blood glucose level post AAV8 injection. (**Bi–iii**) Percentage increase in body weight post AAV8 injection. Data was presented as mean±s.e.m. *n* = 5. (**Ci**–**iii**) Corresponding AAV genome copies per liver cell at endpoint. AAV genome copies per liver cell for surviving mice in 2 × 10^9^ vg/mouse of AAV8-R3GHLP-hINSco-e(+) was excluded because only 1 mouse survived till the endpoint due to severe hyperglycemia. Mice that were found dead in the cage before endpoint were also excluded. Statistical significances for (**A**,**B**) were determined by two-way ANOVA where ns indicated not significant, ** indicates *p* < 0.01 and considered very significant and **** indicates *p* < 0.0001 and considered extremely significant. Statistical significances for (**C**) were determined by Student’s unpaired *t*-test where *** indicates *p* < 0.001 and considered extremely significant.

To evaluate the potential of R3GHLP promoter coupled with 3′iALB enhancer in vivo, AAV8-R3GHLP-hINSco-e(+) was generated. The AAV8-R3GHLP-hINSco-e(+) was systemically injected into STZ-induced diabetic mice were compared to AAV8-HLP-hINSco with 2 × 10^9^ (Figure 6Ai) and 10 × 10^9^ (Figure 6Aii) vg/mouse.

The mice injected with AAV8-R3GHLP-hINSco-e(+) did not restore to normoglycemia at 10 × 10^9^. We subsequently showed that 20 × 10^9^ vg/mouse of AAV8-R3GHLP-hINSco-e(+) was needed to effectively normalize the blood glucose (Figure 6Aiii). This dose was twice the amount of AAV8-HLP-hINSco (10 × 10^9^ vg/mouse; Figure 3Aiii and Figure 6Aii) and 4 times more of AAV8-R3GHLP-hINSco (5 × 10^9^ vg/mouse; Figure 3Ai). The corresponding body weight was shown in Figure 6Bi–iii.

Further examination of the corresponding AAV genome copies per liver cell revealed that AAV8-R3GHLP-hINSco-e(+) showed very low gene delivery efficiency after systemic delivery (Figure 6Ci–iii). At 10 × 10^9^ vg/mouse dose, genome copies of AAV8-R3GHLP-hINSco-e(+) was 45-fold lower than AAV8-HLP-hINSco even though the AAV8 were administrated at same dose (Figure 6Cii). At 20 × 10^9^ vg/mouse effective dose of AAV8-R3GHLP-hINSco-e(+), as low as 0.085 AAV genome copies were detected in a liver cell (Figure 6Ciii). This was 10.2 to 18.3-fold and 4.7-fold lower when compared to other effective doses in mice injected with 10 × 10^9^ vg/mouse of AAV8-HLP-hINSco (Figure 3Ciii and Figure 6Cii) and 5 × 10^9^ vg/mouse of AAV8-R3GHLP-hINSco (Figure 3Ci), respectively. These results clearly suggested that 3′iALB could further improve the efficacy of R3GHLP promoter if the delivery efficacy was not compromised. Further studies are needed to investigate why AAV8-R3GHLP-hINSco-e(+) showed inefficient gene delivery, which is beyond the scope of this study.

## 4. Discussion

Rapid glucose responsive insulin release into the blood circulation is desirable in the treatment of diabetes. The understanding of the mechanisms of glucose responsive gene expression [11,12,13,14,16,18,26] enables researchers to transform the promoter of interest to being glucose responsive by incorporating the R3G glucose response elements. The rational of using 3 copies of GlRE in R3G was demonstrated by various studies [27,28,29,30] whereas the use of GlRE in reverse orientation was based on work done by Thule and colleagues [28].

We have previously reported the normalization of blood glucose of diabetic mice treated with AAV8 insulin gene therapy using a strong liver specific promoter [5,6,7]. In this study, we incorporate the R3G adjacent to the strong liver-specific HLP promoter. We hypothesized that this will improve the efficacy of the treatment of diabetes when compared to the constitutive HLP promoter.

The in vitro studies showed that in the pR3GHLP-NlucP transfected rHeps, the NlucP gene expression was increased in response to increasing glucose concentration (Figure 1B). We found that glucose responsiveness was achieved via an increase in promoter activity (Figure 1F), presumably by the recruitment of more transcription factors that bind to GlRE. Due to the stronger R3GHLP promoter activity in diabetic blood glucose when compared to HLP promoter, AAV8-R3GHLP-hINSco required less dose at 5 × 10^9^ vg/mouse to treat the diabetic mice (Figure 3Ai).

To date, several groups have generated glucose responsive promoters with the incorporation of GlREs. When compared between the fold increases of gene expression in vitro between cells exposed to <5.5 mM versus >20 mM glucose, the extent of the glucose responsiveness varied across different promoters. For instance, glucose responsiveness was in the range of 2 to 3-fold increase when GlRE was coupled with LPK [29,31], IGFBP1 [28,32], or G6Pase [30] promoters. The best reported glucose responsiveness was observed with albumin promoter with 8 to 9-fold increase of gene expression level [19,27]. In this study, when tested in rHeps, glucose responsiveness between 5.5 mM versus 25 mM glucose of R3GHLP promoter were in range of 20.9 to 58.9-fold when using the NlucP as reporter gene (Figure 1B,F and Figure 5) compared to R3GhLPK 2.6-fold (Figure 1C) and R3GrLPK 8.5-fold (Figure 1D). We chose to test the glucose responsive promoter in rHeps to better represent the normal hepatocytes. The difference in glucose responsiveness fold was probably due to batch to batch variation of the isolated primary cells rHeps used.

The high fold of glucose responsiveness of glucose responsive promoter may not sufficient for treatment of type I diabetes as insulin expression may require strict control and regulation [33]. For example, if the expression of the insulin is too low, the hyperglycemia remained. On the other hand, when the expression level of insulin is too high, the risk of hypoglycemia may also increase. Therefore, optimum expression of insulin is required to have normoglycemic control of blood glucose level. Unlike beta cells that could release the insulin from granules to rapidly reduce the high glucose level in blood, glucose responsive promoter suffered from long lag time to increase production of insulin (kinetic study in Supplementary Figure S3B). This was also demonstrated in IPGTT in Figure 4B, whereby blood glucose rapidly reduced to normal level within 30 min in normal mice (Figure 4Bi), whereas longer time was needed for AAV8-HLP-hINSco (Figure 4Biii) and AAV8-R3GHLP-hINSco (Figure 4Biv) to normalize the blood glucose. The reduction in blood glucose in IPGTT of AAV8-R3GHLP-hINSco and AAV8-HLP-hINSco is due to the continuous production of insulin driven by both the HLP and R3GHLP promoters. The return of blood glucose level to pre-induction level is slightly faster for the R3GHLP promoter probably due to its higher activity at high glucose level compared to HLP promoter but the increase of R3GHLP promoter activity is gradual and only peaked at 10 h (Supplementary Figure S3B). We conclude that the glucose responsive promoter is not able to mimic fully to cause a burst of insulin secretion in the beta cells in response to high glucose. Nevertheless, it would be a useful promoter to express insulin at a basal level with similar to the commercially available long acting insulin.

On the other hand, diabetic mice treated with AAV8-HLP-hINSco or AAV8-R3GHLP-hINSco consistently showed lower fasting blood glucose levels when compared to fasting blood glucose in normal mice (Figure 4A). Similar to the case of IPGTT, the reduction of the blood glucose during fasting of AAV8-HLP-hINSco or AAV8-R3GHLP-hINSco treated mice is also due to continuous production of insulin with HLP constitutive promoter and R3GHLP glucose responsive promoter. Despite the capability of glucose responsive dependent gene expression with R3GHLP promoter, long lag time for this promoter to reduce its expression level as shown in Supplementary Figure S3B had led to continuously production of human insulin in liver causing the lower fasting blood glucose when compared with normal mice.

The incorporation of 3′iALB at 3′end of human insulin gene was shown to increase the expression of insulin level for about 6-fold in adenovirus transduced freshly isolated rHeps culture in complete medium containing 25 mM glucose [19]. In the liver cells, Wooddell and colleagues showed that intron 14 in 3′iALB could further improve the expression level for another 5-fold when compared to human albumin 3′UTR without intron [20]. When 3′iALB was incorporated at 3′end of reporter gene in pR3GHLP-NlucP in this study, we observed 7.5-fold increase in promoter activity at 25 mM glucose when compared to expression level of pR3GHLP-NlucP without 3′iALB (Figure 5). Additionally, the glucose responsiveness between 5.5 mM versus 25 mM glucose was also further increased to 169-fold.

Surprisingly, when AAV8-R3GHLP-hINSco and AAV8-R3GHLP-hINSco-e(+) were injected into diabetic mice, 4 times more AAV8-R3GHLP-hINSco-e(+) (Figure 6Aiii, 20 × 10^9^ vg/mouse) was required to achieve similar blood glucose outcome as AAV8-R3GHLP-hINSco (without 3′iALB; Figure 3Ai, 5 × 10^9^ vg/mouse). Analysis of liver samples at endpoint indicated that 20 × 10^9^ vg/mouse AAV8-R3GHLP-hINSco-e(+) (0.085 ± 0.027 AAV genome copies/liver cell; Figure 6Ciii) has fewer viral genome copies/liver cell compared to 5 × 10^9^ vg/mouse AAV8-R3GHLP-hINSco (0.401 ± 0.046 AAV genome copies/liver cell; Figure 3Ci). Although higher dose was required to normalize blood glucose with AAV8-R3GHLP-hINSco-e(+), the amount of transgene that was successfully delivered into the liver cells was only 0.085 copies per liver cell (Figure 6Ciii). In other words, 0.085 copies per liver cell of AAV8-R3GHLP-hINSco-e(+) with 3′iALB was sufficient to produce enough insulin for blood glucose normalization. On the other hand, the result from Figure 3 indicated that 0.40 copies per liver cells of AAV8-R3GHLP-hINSco without 3′iALB (Figure 3Ci) was needed to produce sufficient insulin for blood glucose normalization (Figure 3Ai). Taken together, AAV8-R3GHLP-hINSco-e(+) produced insulin with higher expression level (about 4.7-fold) compared to AAV8-R3GHLP-hINSco.

We hypothesized that the reduction in AAV8-R3GHLP-hINSco-e(+)genome copies per liver cell may be caused by (1) higher transgene size lowering liver transduction efficacy in vivo (2) the 3iALB affecting the AAV genome stability in liver cells, different from when it was in a plasmid setting in vivo, or (3) unknown mechanisms. The insertion of an 860 bp fragment containing 3′iALB increased the size of L-ITR to R-ITR of AAV8-R3GHLP-hINSco from 1986 bp to 2846 bp. We observed similar negative correlation between the AAV8 genome sizes versus in vivo AAV genome copies per liver cell (observation when comparing results across various experiments performed in our laboratory). To the best of our knowledge, no studies had reported the effect of the AAV8 genome size on it gene delivery in vivo, and we hereby caution the use of AAV8 vectors with larger insert size even if the in vitro transfection results showed superiority in expression.

In this study, we converted a strong constitutive liver specific promoter to one that is glucose responsive by inserting glucose responsive elements to the 5′ end. The glucose responsiveness is via the amplification of its gene expression in high glucose environment. Less viral load would be required to bring down the high blood glucose level in diabetic patients compared to the constitutive liver-specific HLP promoter thereby reducing the risk of liver toxicity or injury [34] and/or activation of AAV8-capsid T cells [35]. The long lag time needed to reach the peak expression upon glucose challenge and the equally long time required for attenuation of the promoter upon glucose withdrawal of such glucose responsive promoters did not match the instantaneously release of insulin by the pancreas. However, the current system, coupled with a safety switch for turning off of insulin secretion under adverse situations [36] would be suitable for long term expression of basal level of insulin using only a one-time injection of virus, which can achieve similar outcome to that of repeated injections of long acting insulin. While the strategy of using a one-time administration of AAV8-R3GHLP-hINSco may still require multiple daily injections of rapid/short-acting insulin to reduce post-meal glucose excursions [37], it may simulate the endogenous constitutive insulin secretion more effectively than the long/ultra-acting insulin, thereby reducing glycemic variability [4,38]. Further research to design strategies to store the excess insulin in hepatocytes and secrete it upon induction by glucose challenge [39,40] may potentially help to overcome the limitations of the current glucose responsive promoters.

## Supplementary Materials

The following are available online at https://www.mdpi.com/2073-4409/9/11/2474/s1, Figure S1: Schematic diagram of cloning for plasmids used in this study; Figure S2: Detail body weight and blood glucose profiles of mouse 3 in AAV8-HLP-hINSco (7.5 × 10^9^) injected group compared to mouse 1 and 2 in the same group; Figure S3: Functionality assessment of 3′ albumin enhancer (3′iALB) in AAV plasmid vector with R3GHLP glucose responsive promoter and firefly luciferase as reporter gene for its enhanced gene expression and glucose responsiveness.

## Abbreviations

AAV	adeno-associated virus
AAV8	AAV serotype 8
HLP	hybrid liver-specific promoter
3′UTR	3′ untranslated region
3′iALB	3′UTR of albumin gene with intron 14
GlRE	glucose responsive element
R3G	3 copies of GlRE in reverse orientation
bp	base pair
STZ	streptozotocin
ChoRE	carbohydrate response element
ChREBP	carbohydrate response element binding protein
LPK	liver pyruvate kinase
hINSco	codon optimized furin cleavable human proinsulin
rHeps	primary rat hepatocytes
WEM	Williams’ E Medium
NSG	NOD.cg-PrkdcscidIl2rgtm1Wjl/SzJ
IPGTT	intraperitoneal glucose tolerant test
s.e.m.	standard error of mean
ns	not significant
NlucP	PEST-destabilized nanoLuc luciferase
vg	vector genome
Fluc	firefly luciferase
ITR	inverted terminal repeat
