# Supplementary material for: Modification of a Constitutive to Glucose-Responsive Liver-Specific Promoter Resulted in Increased Efficacy of Adeno-Associated Virus Serotype 8-Insulin Gene Therapy of Diabetic Mice
Source: Cells. 2020 Nov 13;9(11):2474. doi: 10.3390/cells9112474 (PMC7696068; doi:10.3390/cells9112474)
Supplement: Supplementary file 1 [file cells-09-02474-s001.pdf]

cells

MDPI

A

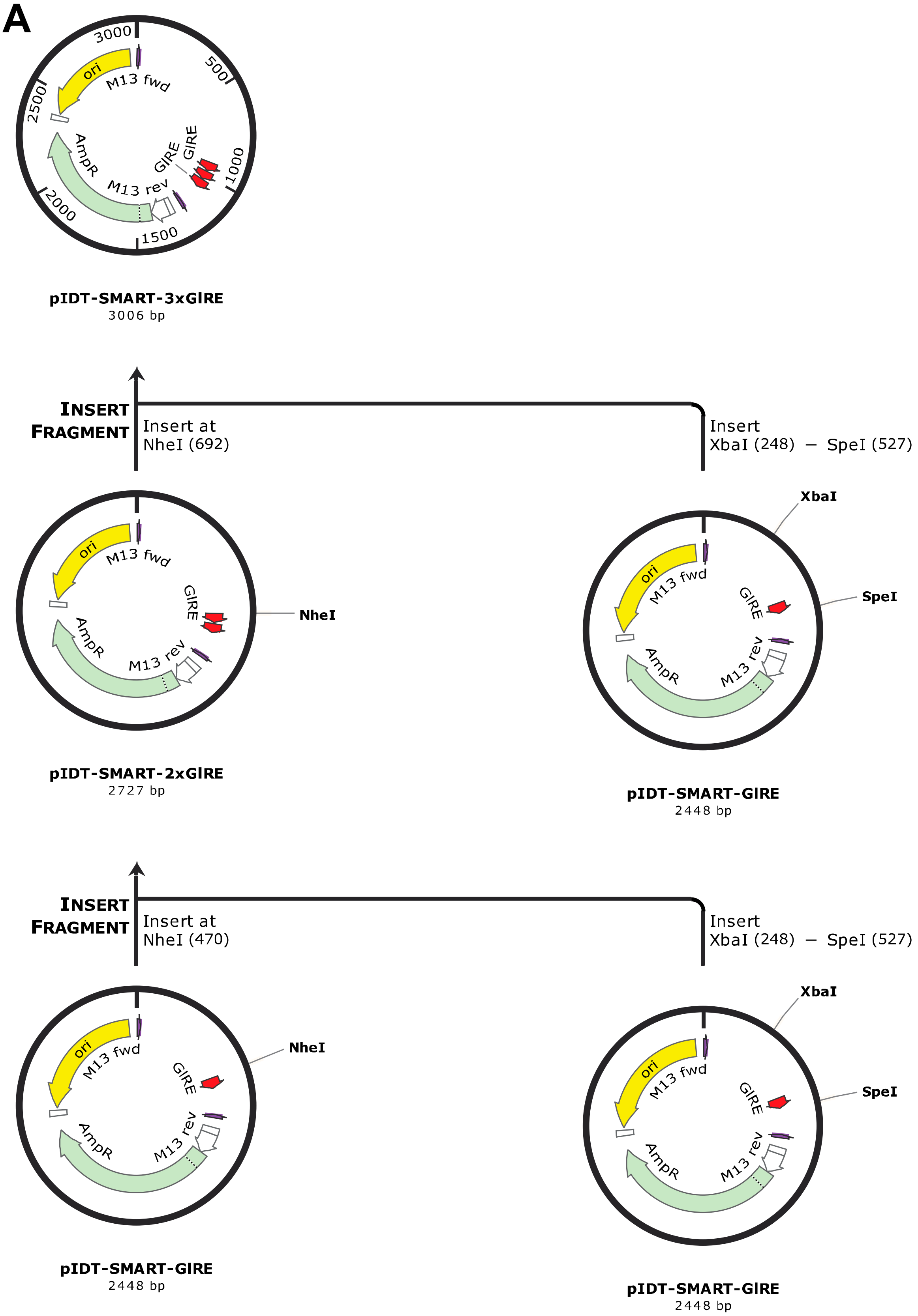
3000
500
1000
1500
2000
2500
ori
M13 fwd
GIRE
GIRE
AmpR
M13 rev
pIDT-SMART-3xGIRE
3006 bp
INSERT FRAGMENT
Insert at NheI (692)
Insert XbaI (248) – SpeI (527)
ori
M13 fwd
GIRE
AmpR
M13 rev
NheI
pIDT-SMART-2xGIRE
2727 bp
XbaI
SpeI
ori
M13 fwd
GIRE
AmpR
M13 rev
pIDT-SMART-GIRE
2448 bp
INSERT FRAGMENT
Insert at NheI (470)
Insert XbaI (248) – SpeI (527)
ori
M13 fwd
GIRE
AmpR
M13 rev
NheI
pIDT-SMART-GIRE
2448 bp
XbaI
SpeI
ori
M13 fwd
GIRE
AmpR
M13 rev
pIDT-SMART-GIRE
2448 bp

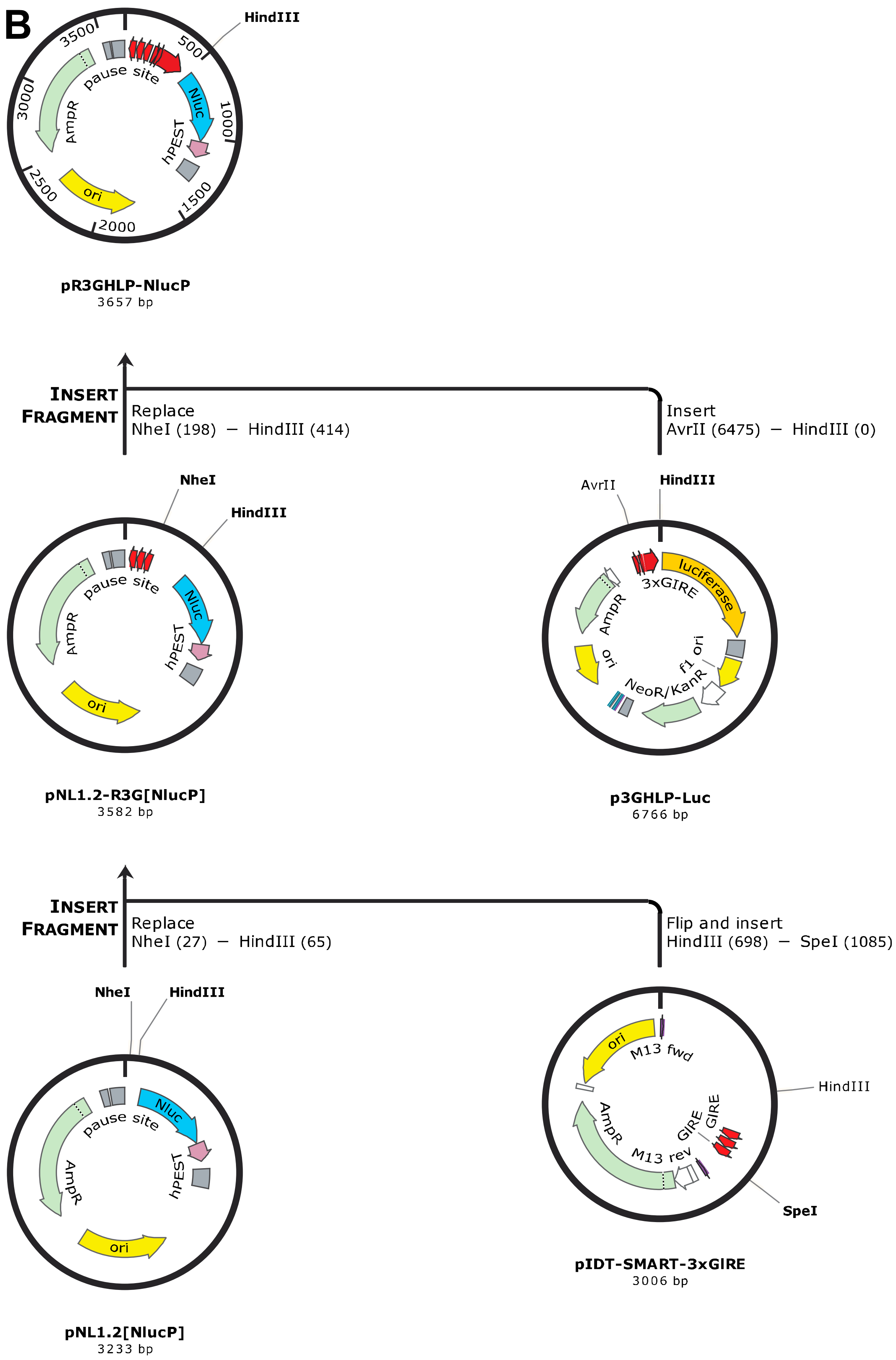
B
HindIII
3500
500
1000
1500
2000
2500
3000
pause site
Nluc
hPEST
AmpR
ori
pR3GHLP-NlucP
3657 bp
INSERT FRAGMENT
Replace
NheI (198) — HindIII (414)
Insert
AvrII (6475) — HindIII (0)
NheI
HindIII
pause site
Nluc
hPEST
AmpR
ori
AvrII
HindIII
3xGIRE
luciferase
AmpR
ori
f1 ori
NeoR/KanR
pNL1.2-R3G[NlucP]
3582 bp
p3GHLP-Luc
6766 bp
INSERT FRAGMENT
Replace
NheI (27) — HindIII (65)
Flip and insert
HindIII (698) — SpeI (1085)
NheI
HindIII
pause site
Nluc
hPEST
AmpR
ori
ori
M13 fwd
HindIII
AmpR
GIRE
GIRE
M13 rev
SpeI
pIDT-SMART-3xGIRE
3006 bp
pNL1.2[NlucP]
3233 bp

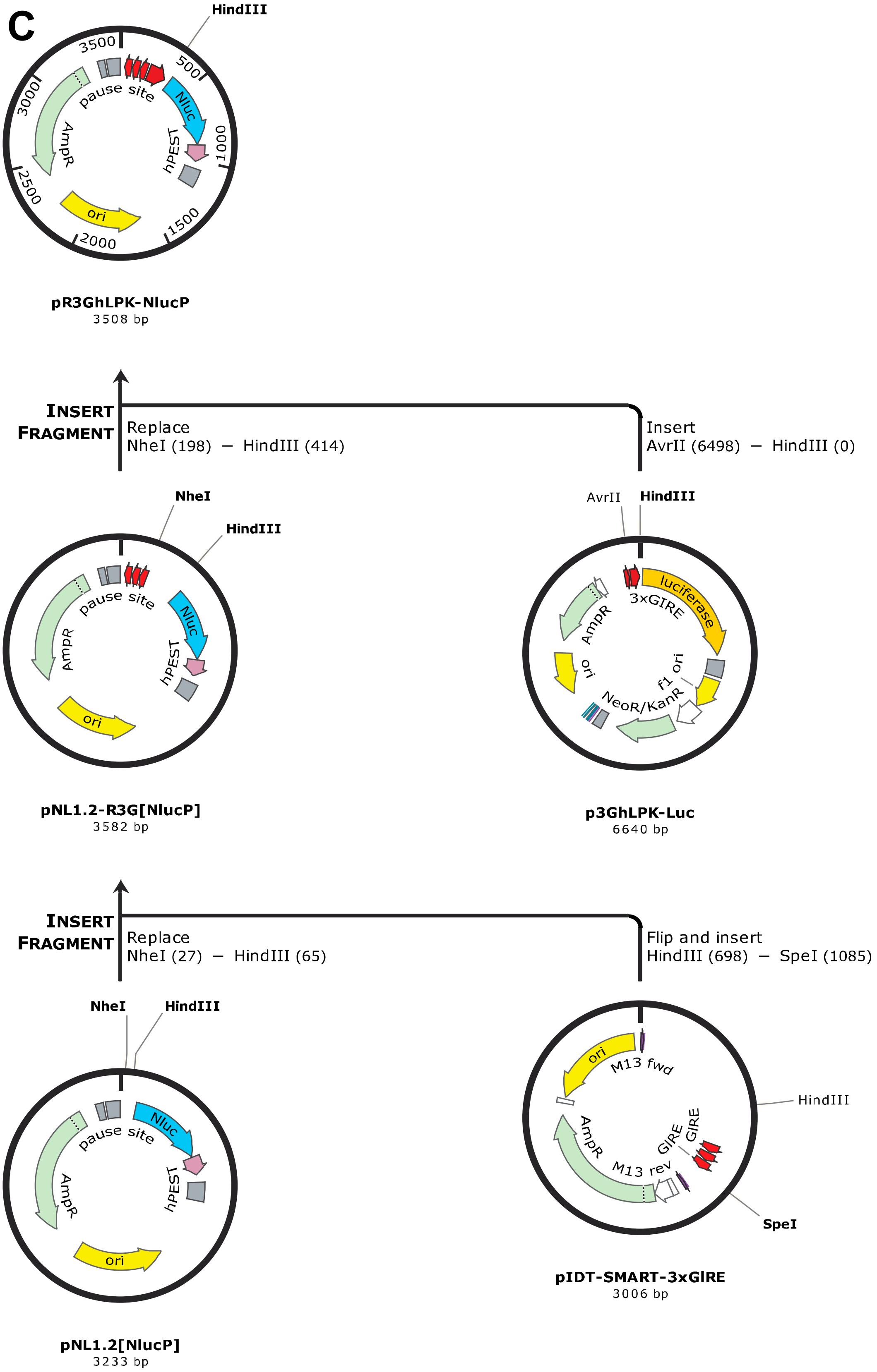
C
HindIII
3500
500
3000
1000
2500
1500
2000
pause site
Nluc
AmpR
hPEST
ori
pR3GhLPK-NlucP
3508 bp
INSERT FRAGMENT
Replace
NheI (198) — HindIII (414)
Insert
AvrII (6498) — HindIII (0)
NheI
HindIII
AvrII
HindIII
pause site
Nluc
AmpR
hPEST
ori
3xGIRE
luciferase
f1 ori
NeoR/KanR
pNL1.2-R3G[NlucP]
3582 bp
p3GhLPK-Luc
6640 bp
INSERT FRAGMENT
Replace
NheI (27) — HindIII (65)
Flip and insert
HindIII (698) — SpeI (1085)
NheI
HindIII
pause site
Nluc
AmpR
hPEST
ori
M13 fwd
GIRE
GIRE
M13 rev
HindIII
SpeI
pIDT-SMART-3xGIRE
3006 bp
pNL1.2[NlucP]
3233 bp

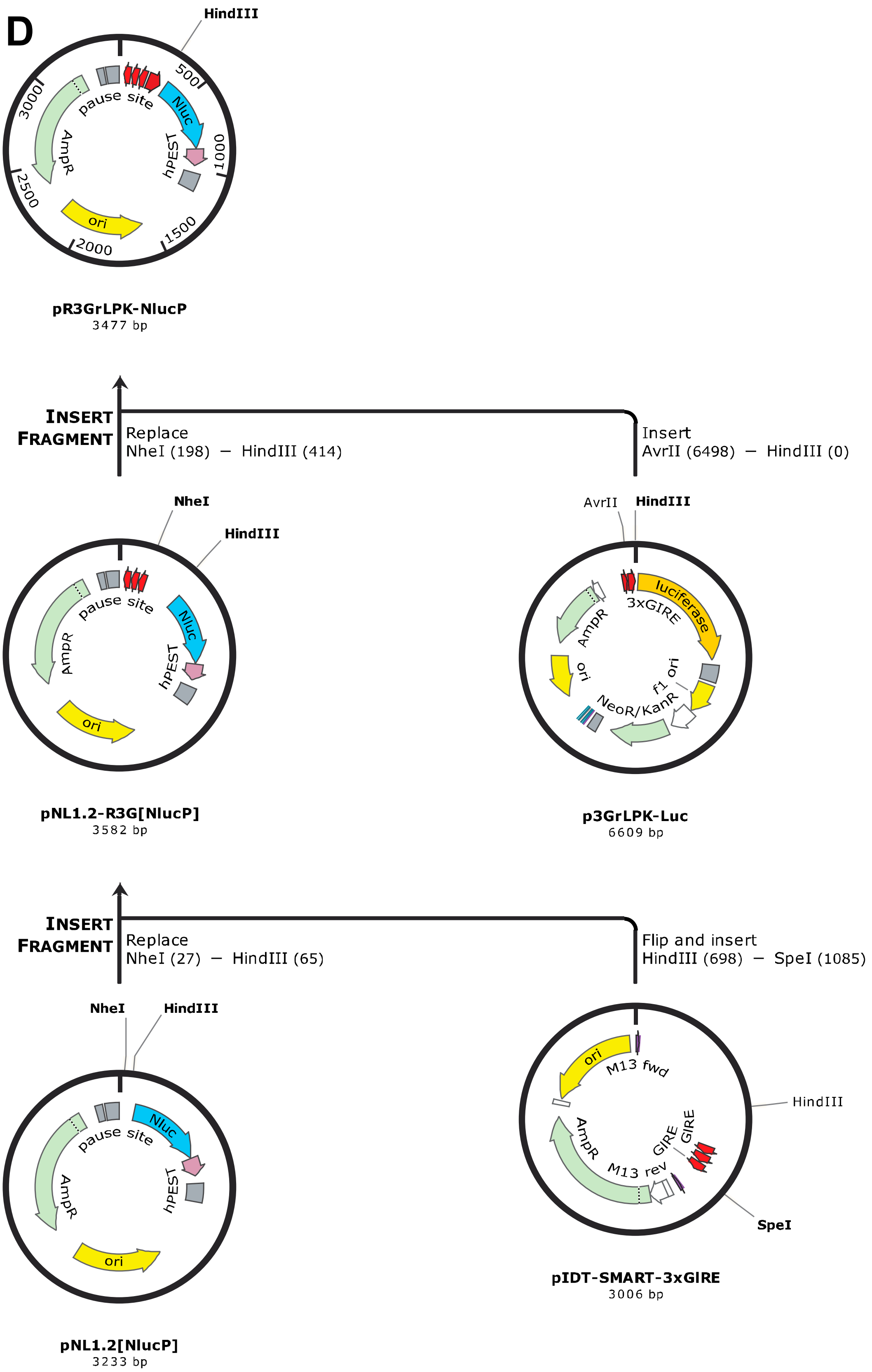
D
HindIII
500
1000
1500
2000
2500
3000
pause site
Nluc
hPEST
AmpR
ori
pR3GrLPK-NlucP
3477 bp
INSERT FRAGMENT
Replace
NheI (198) – HindIII (414)
Insert
AvrII (6498) – HindIII (0)
NheI
HindIII
pause site
Nluc
hPEST
AmpR
ori
pNL1.2-R3G[NlucP]
3582 bp
AvrII
HindIII
3xGIRE
luciferase
AmpR
ori
f1 ori
NeoR/KanR
p3GrLPK-Luc
6609 bp
INSERT FRAGMENT
Replace
NheI (27) – HindIII (65)
Flip and insert
HindIII (698) – SpeI (1085)
NheI
HindIII
pause site
Nluc
hPEST
AmpR
ori
pNL1.2[NlucP]
3233 bp
ori
M13 fwd
AmpR
GIRE
GIRE
M13 rev
HindIII
SpeI
pIDT-SMART-3xGIRE
3006 bp

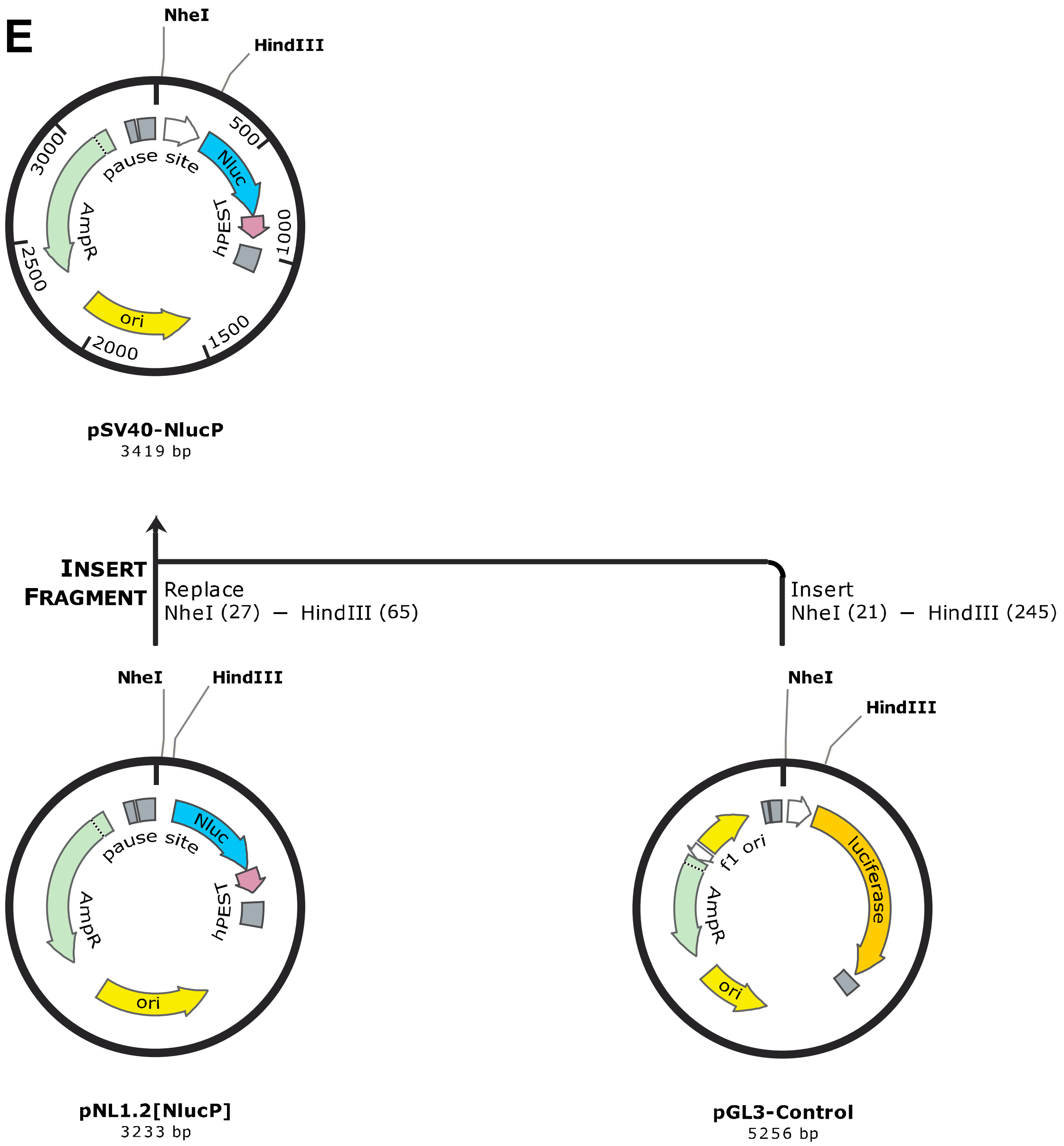
E
NheI
HindIII
500
1000
1500
2000
2500
3000
pause site
Nluc
hPEST
AmpR
ori
pSV40-NlucP
3419 bp
INSERT
FRAGMENT
Replace
NheI (27) — HindIII (65)
Insert
NheI (21) — HindIII (245)
NheI
HindIII
pause site
Nluc
hPEST
AmpR
ori
pNL1.2[NlucP]
3233 bp
NheI
HindIII
f1 ori
luciferase
AmpR
ori
pGL3-Control
5256 bp

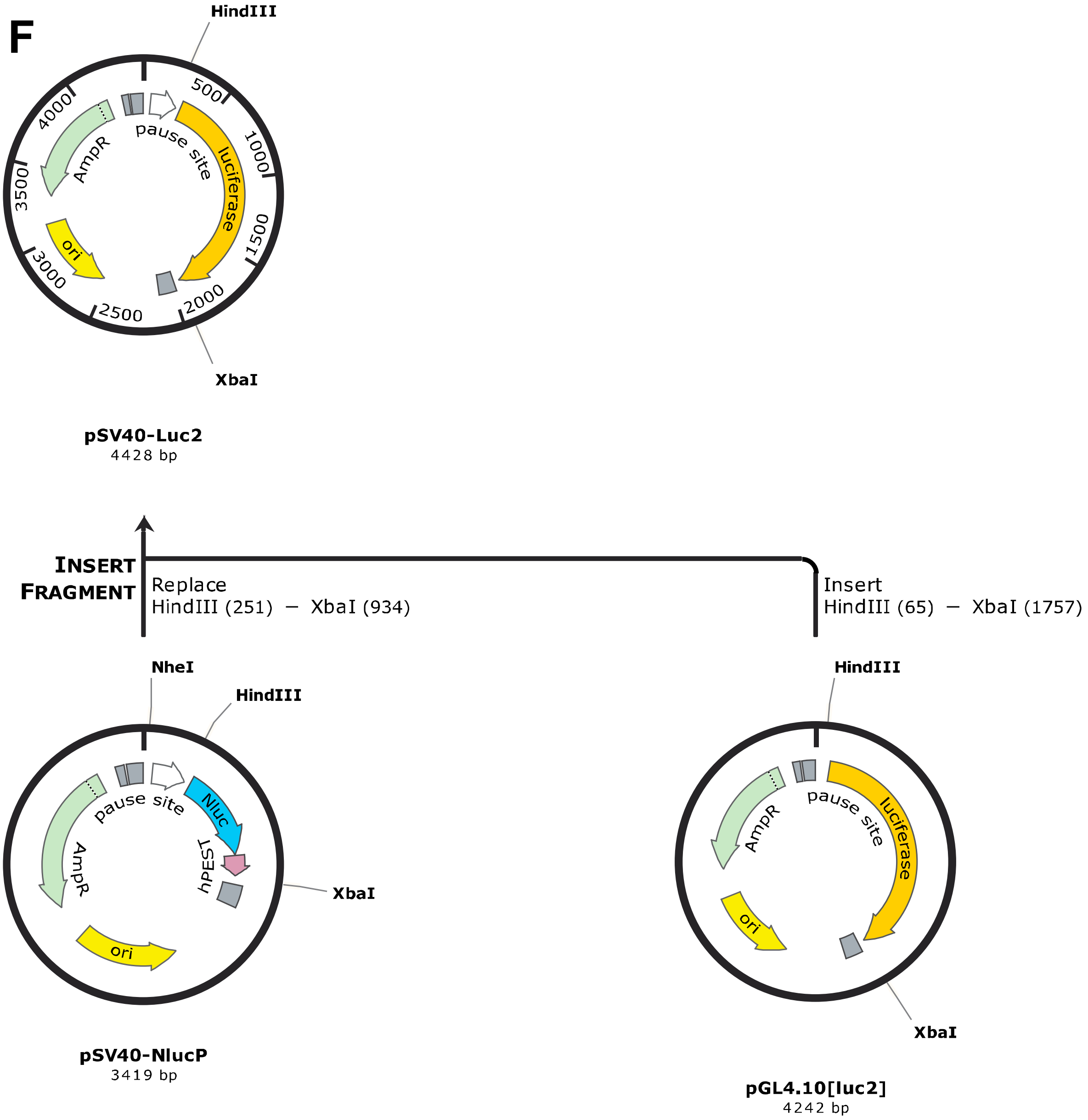
F
HindIII
500
1000
1500
2000
2500
3000
3500
4000
AmpR
pause site
luciferase
ori
XbaI
pSV40-Luc2
4428 bp
INSERT
FRAGMENT
Replace
HindIII (251) – XbaI (934)
Insert
HindIII (65) – XbaI (1757)
NheI
HindIII
pause site
Nluc
hPEST
AmpR
ori
XbaI
pSV40-NlucP
3419 bp
HindIII
AmpR
pause site
luciferase
ori
XbaI
pGL4.10[luc2]
4242 bp

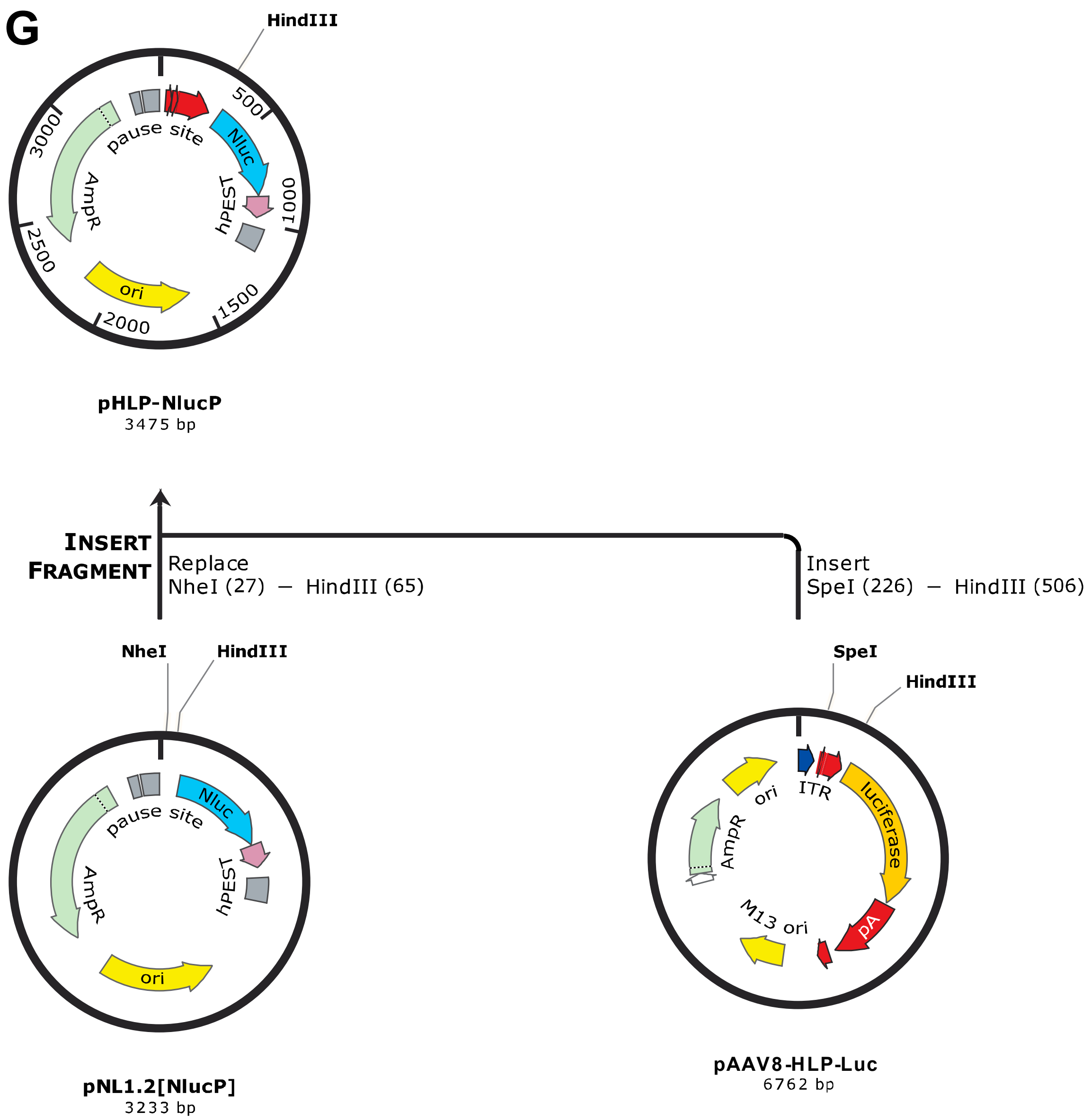
G
HindIII
pause site
Nluc
hPEST
AmpR
ori
500
1000
1500
2000
2500
3000
pHLP-NlucP
3475 bp
INSERT
FRAGMENT
Replace
NheI (27) — HindIII (65)
Insert
SpeI (226) — HindIII (506)
NheI
HindIII
pause site
Nluc
hPEST
AmpR
ori
pNL1.2[NlucP]
3233 bp
SpeI
HindIII
ITR
luciferase
ori
AmpR
M13 ori
pA
pAAV8-HLP-Luc
6762 bp

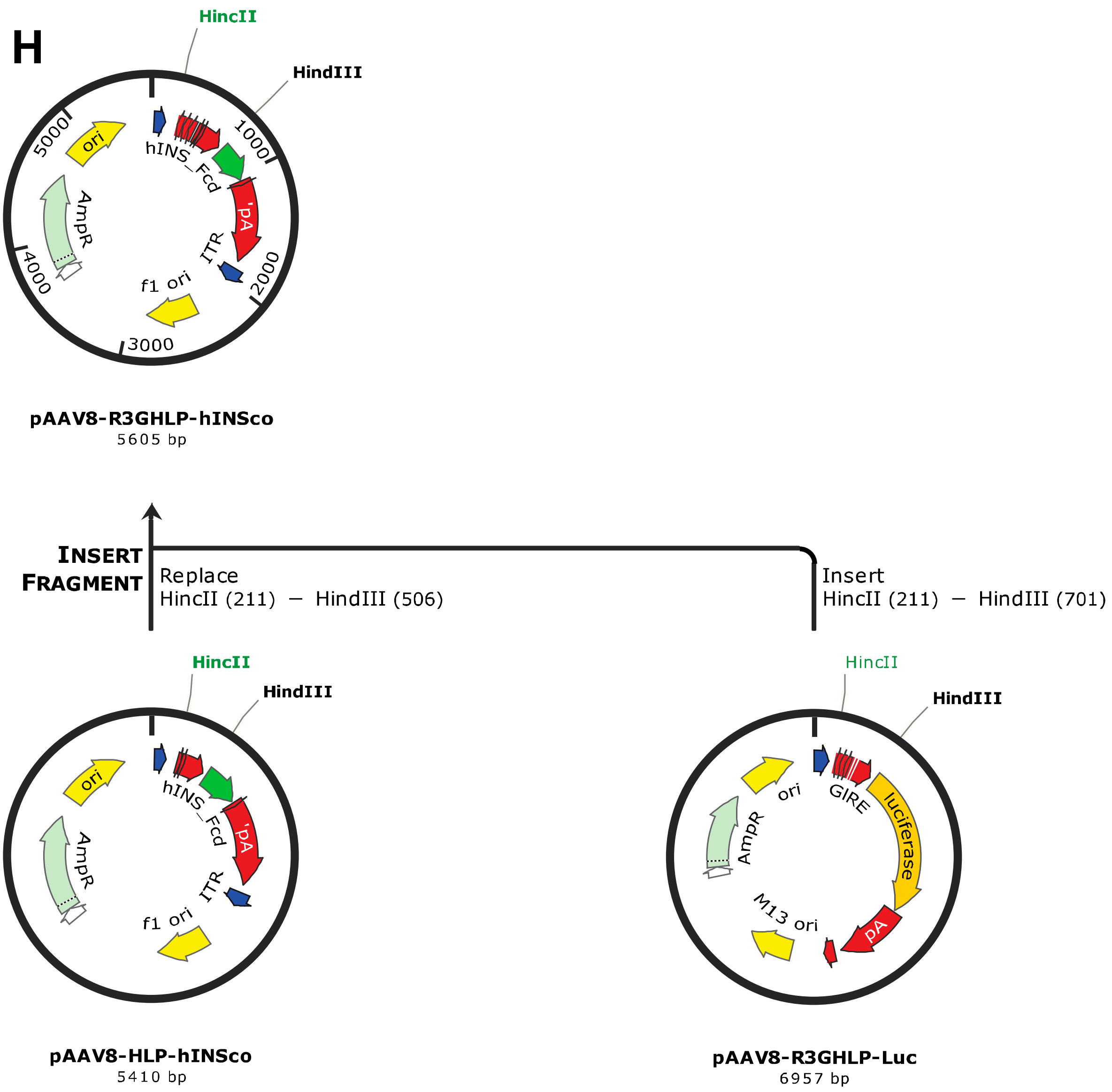
H
HincII
HindIII
1000
2000
3000
4000
5000
ori
hINS_Fcd
'pA
ITR
f1 ori
AmpR
pAAV8-R3GHLP-hINSco
5605 bp
INSERT FRAGMENT
Replace
HincII (211) — HindIII (506)
Insert
HincII (211) — HindIII (701)
HincII
HindIII
ori
hINS_Fcd
'pA
ITR
f1 ori
AmpR
pAAV8-HLP-hINSco
5410 bp
HincII
HindIII
ori
GIRE
luciferase
AmpR
M13 ori
pA
pAAV8-R3GHLP-Luc
6957 bp

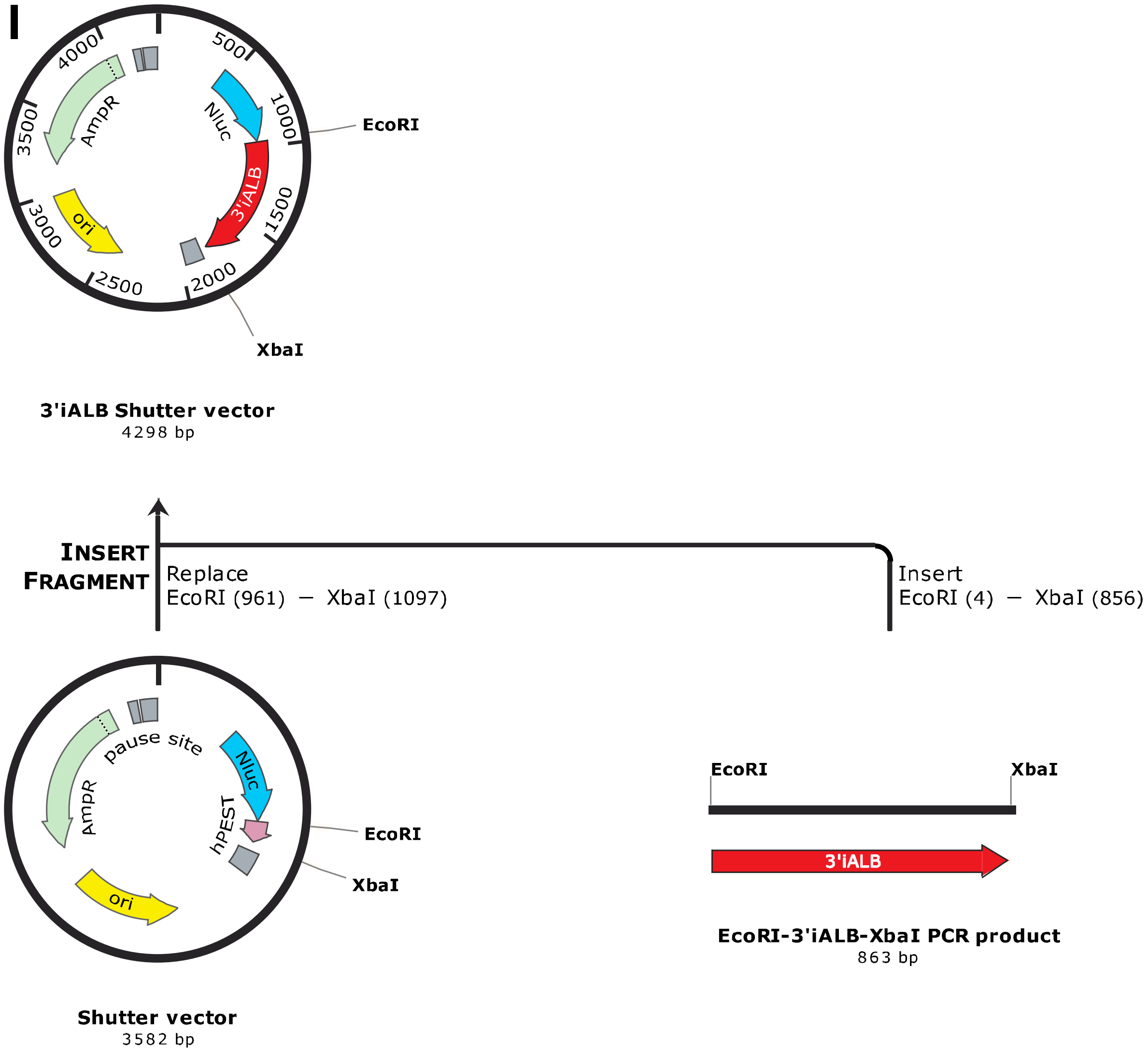
I
500
1000
1500
2000
2500
3000
3500
4000
Nluc
3'iALB
AmpR
ori
EcoRI
XbaI
3'iALB Shutter vector
4298 bp
INSERT FRAGMENT
Replace
EcoRI (961) — XbaI (1097)
Insert
EcoRI (4) — XbaI (856)
pause site
Nluc
hPEST
AmpR
ori
EcoRI
XbaI
Shutter vector
3582 bp
EcoRI
XbaI
3'iALB
EcoRI-3'iALB-XbaI PCR product
863 bp

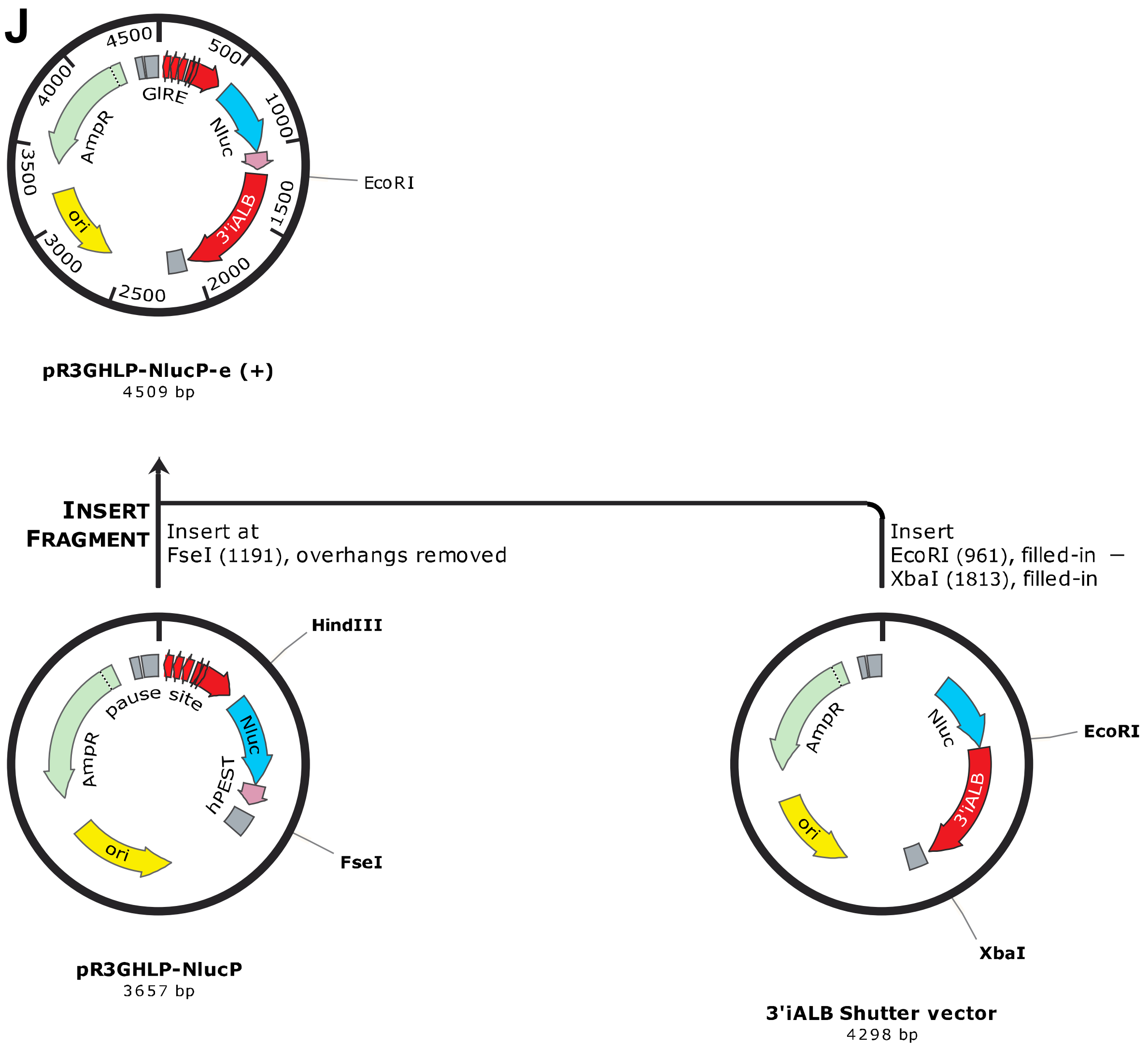
J
4500
500
1000
1500
2000
2500
3000
3500
4000
GIRE
Nluc
AmpR
ori
3'iALB
EcoRI
pR3GHLP-NlucP-e (+)
4509 bp
INSERT FRAGMENT
Insert at
FseI (1191), overhangs removed
Insert
EcoRI (961), filled-in –
XbaI (1813), filled-in
HindIII
pause site
Nluc
AmpR
hPEST
ori
FseI
pR3GHLP-NlucP
3657 bp
AmpR
Nluc
EcoRI
3'iALB
ori
XbaI
3'iALB Shutter vector
4298 bp

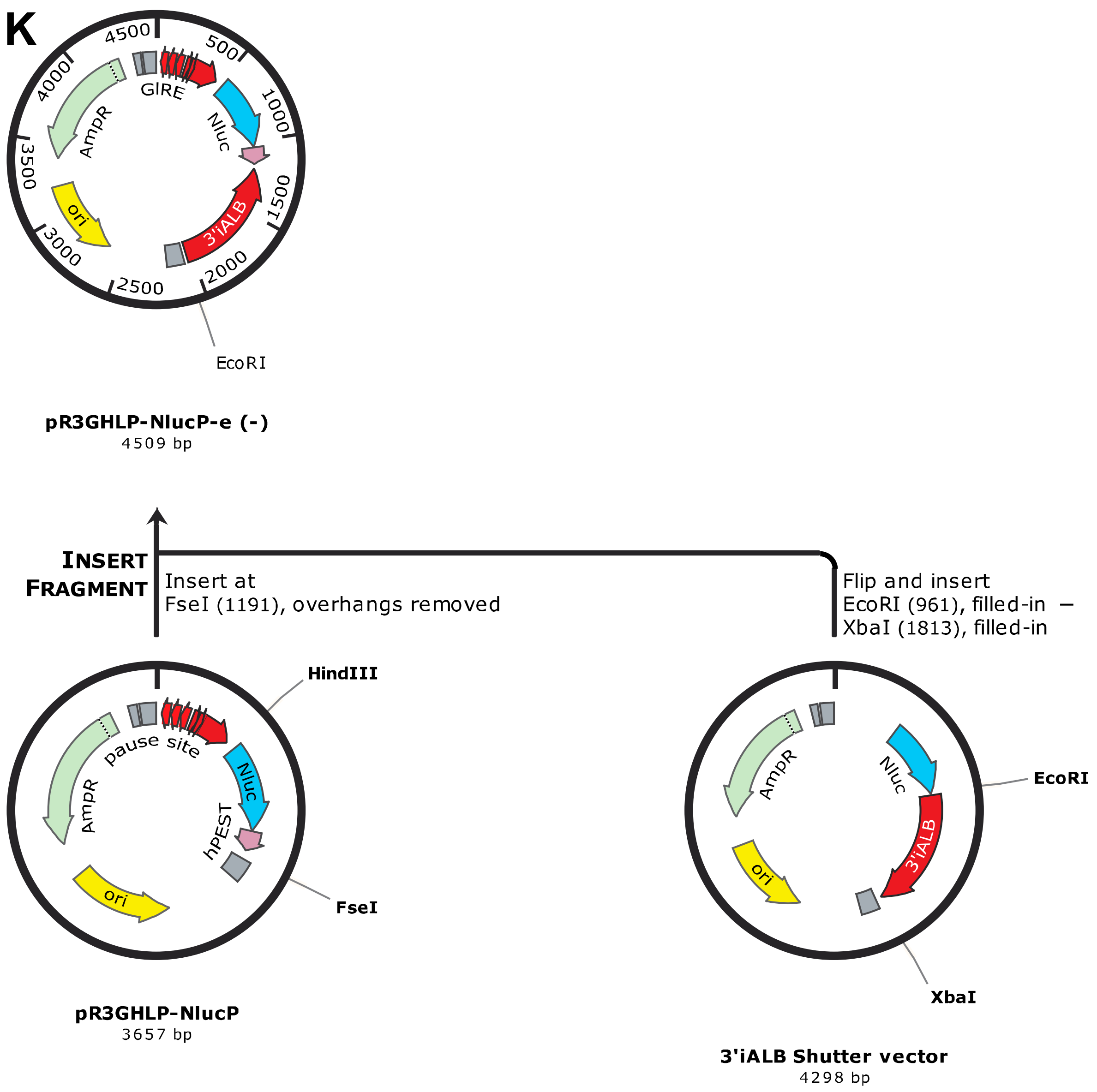
K
4500
500
4000
1000
3500
1500
3000
2500
2000
GIRE
Nluc
AmpR
ori
3'iALB
EcoRI
pR3GHLP-NlucP-e (-)
4509 bp
INSERT FRAGMENT
Insert at
FseI (1191), overhangs removed
Flip and insert
EcoRI (961), filled-in –
XbaI (1813), filled-in
HindIII
pause site
Nluc
AmpR
hPEST
ori
FseI
pR3GHLP-NlucP
3657 bp
AmpR
Nluc
EcoRI
3'iALB
ori
XbaI
3'iALB Shutter vector
4298 bp

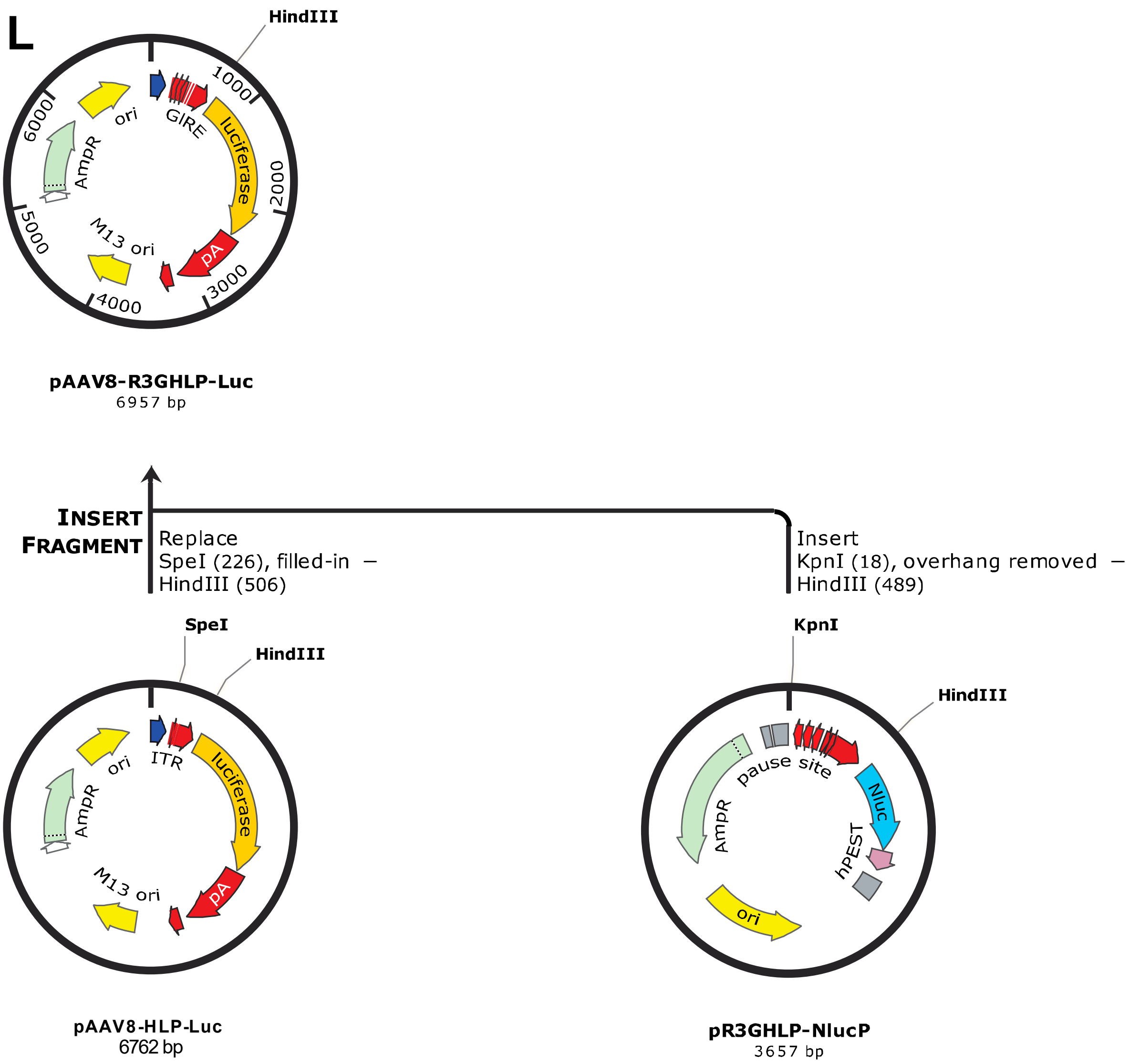
L
HindIII
1000
2000
3000
4000
5000
6000
ori
GIRE
luciferase
AmpR
M13 ori
pA
pAAV8-R3GHLP-Luc
6957 bp
INSERT FRAGMENT
Replace
SpeI (226), filled-in –
HindIII (506)
Insert
KpnI (18), overhang removed –
HindIII (489)
SpeI
HindIII
ori
ITR
luciferase
AmpR
M13 ori
pA
pAAV8-HLP-Luc
6762 bp
KpnI
HindIII
pause site
Nluc
AmpR
hPEST
ori
pR3GHLP-NlucP
3657 bp

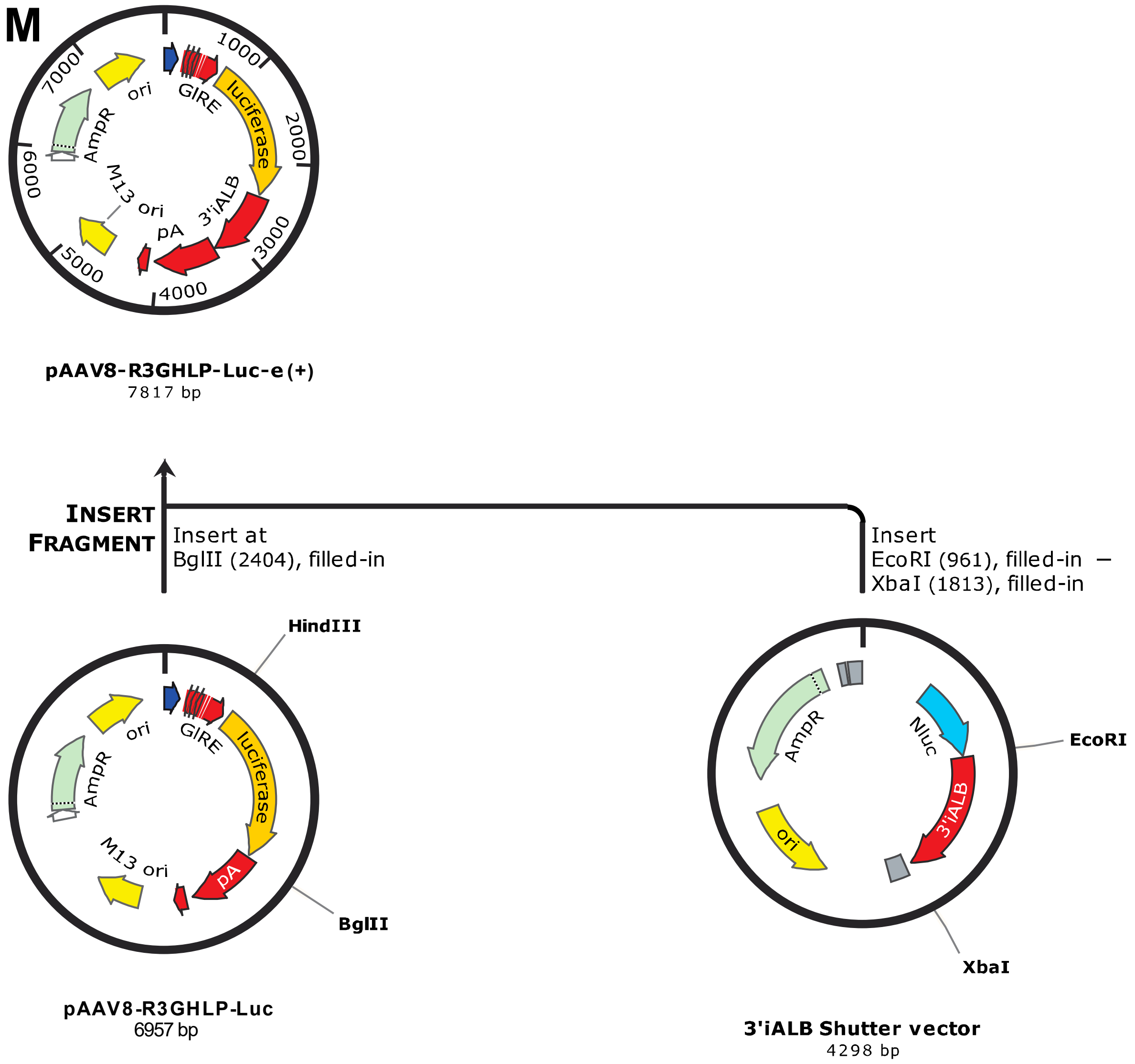
M
ori
GlRE
luciferase
AmpR
M13 ori
pA
3'iALB
1000
2000
3000
4000
5000
6000
7000
pAAV8-R3GHLP-Luc-e(+)
7817 bp
INSERT FRAGMENT
Insert at
BglII (2404), filled-in
Insert
EcoRI (961), filled-in –
XbaI (1813), filled-in
HindIII
BglII
EcoRI
XbaI
NLuc
pAAV8-R3GHLP-Luc
6957 bp
3'iALB Shutter vector
4298 bp

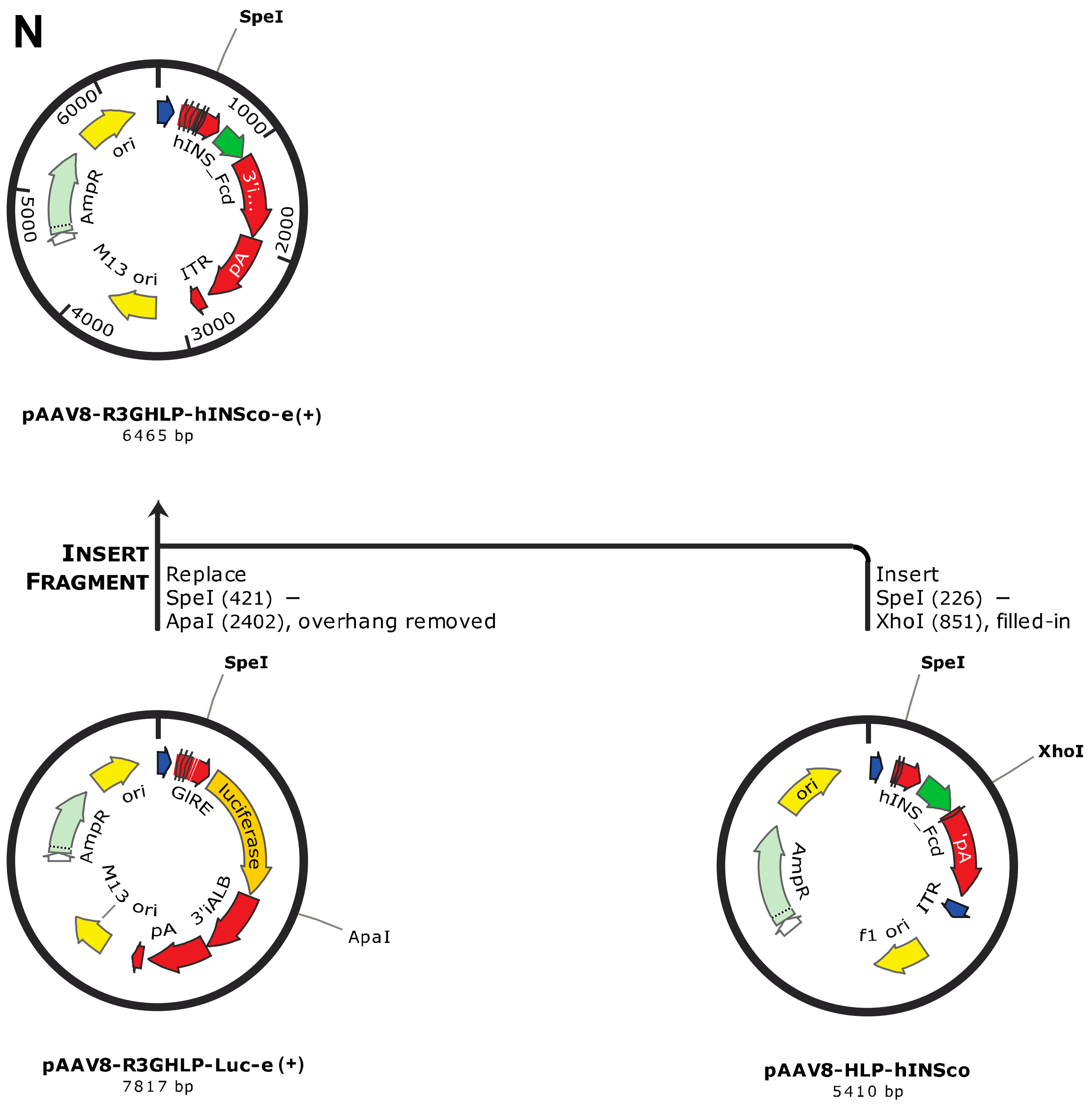

Supplementary Figure S1. Schematic diagram of cloning for plasmids used in this study. (**A**) pIDT-SMART-3xGlRE; (**B**) pR3GHLP-NlucP; (**C**) pR3GhLPK-NlucP; (**D**) pR3GrLPK-NlucP; (**E**) pSV40-NlucP; (**F**) pSV40-Luc2; (**G**) pHLP-NlucP; (**H**) pAAV8-R3GHLP-hINSco; (**I**) 3'iALB shutter vector; (**J**) pR3GHLP-NlucP-e(+); (**K**) pR3GHLP-NlucP-e(-); (**L**) pAAV8-R3GHLP-Luc; (**M**) pAAV8-R3GHLP-Luc-e(+); (**N**) pAAV8-R3GHLP-hINSco-e(+).

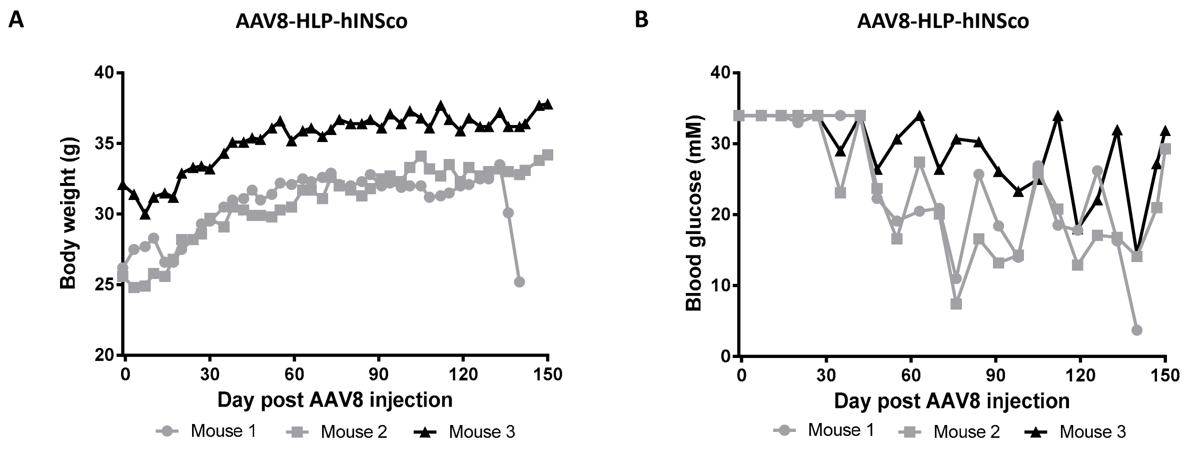

Supplementary Figure S2. Detail body weight and blood glucose profiles of mouse 3 in AAV8-HLP-hINSco (7.5 × 10^9) injected group compared to mouse 1 and 2 in the same group. (**A**) Body weight for individual mouse. (**B**) Blood glucose for individual mouse. Mouse 1 was euthanized before endpoint at 140 days post adeno-associated virus serotype 8 (AAV8) injection as it showed dehydration and more than 20% weight loss according to NUS Institutional Animal Care and Use Committee (IACUC) guidelines.

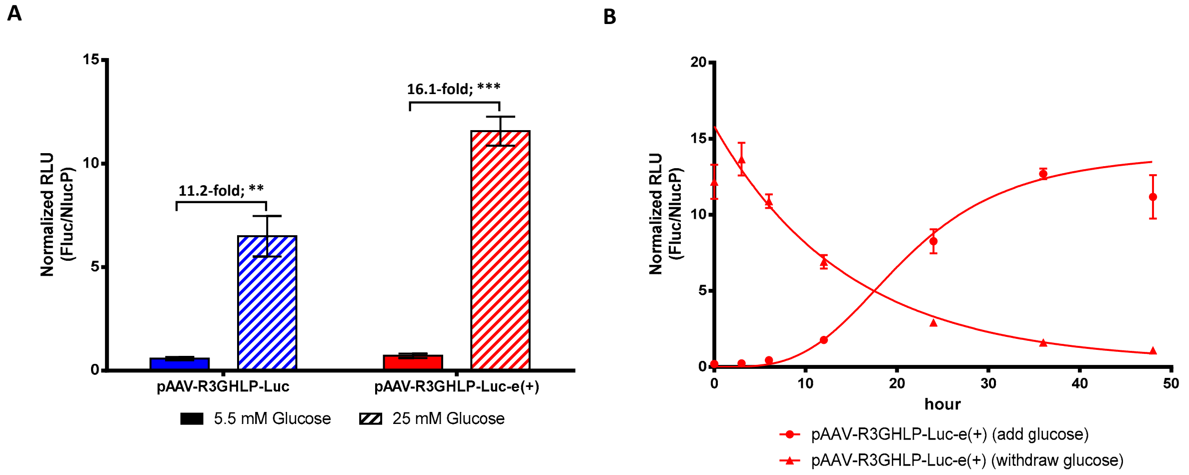

Supplementary Figure S3. Functionality assessment of 3′ albumin enhancer (3′iALB) in AAV plasmid vector with R3GHLP glucose responsive promoter and firefly luciferase as reporter gene for its enhanced gene expression and glucose responsiveness. (**A**) Glucose responsive gene expression level of pAAV-R3GHLP-Luc-e(+)(forward direction only) containing 3′iALB at 5.5 versus 25 mM glucose. The expression was compared to pAAV-R3GHLP-Luc without 3′iALB. $n = 3$. (**B**) Glucose induction and withdrawal effect in pAAV-R3GHLP-Luc-e(+). After overnight transfection, glucose was increase from 5.5 mM to 25 mM in induction group (add glucose) whereas glucose was reduced from 25 mM to 5.5 mM in withdrawal group (withdraw glucose). Luciferase activities were measured at 0, 3, 6, 12, 24, 36, and 48 h, $n = 6$. All experiments were performed in vitro with freshly isolated primary rat hepatocytes culture where plasmids were transfected using lipofectamin 3000. Firefly luciferase (Fluc) was used as reporter gene and the expression levels were normalized to PEST-destabilized nanoLuc luciferase (NlucP) activities from co-transfected pSV40-NlucP constitutively expressed control reporter plasmid. All data were presented as mean ± s.e.m. Statistical significances were determined by Student's unpaired *t*-test where ** indicates $p < 0.01$ and considered very significant and *** indicates $p < 0.001$ and considered extremely significant.
